# Feature Selection Problem and Metaheuristics: A Systematic Literature Review about Its Formulation, Evaluation and Applications

**DOI:** 10.3390/biomimetics9010009

**Published:** 2023-12-25

**Authors:** José Barrera-García, Felipe Cisternas-Caneo, Broderick Crawford, Mariam Gómez Sánchez, Ricardo Soto

**Affiliations:** 1 Escuela de Ingeniería Informática, Pontificia Universidad Católica de Valparaíso, Avenida Brasil 2241, Valparaíso 2362807, Chile; jose.barrera@pucv.cl (J.B.-G.); felipe.cisternas.c@mail.pucv.cl (F.C.-C.); ricardo.soto@pucv.cl (R.S.); 2 Departamento de Electrotecnia e Informática, Universidad Técnica Federico Santa María, Federico Santa María 6090, Viña del Mar 2520000, Chile; mariam.gomez@usm.cl

**Keywords:** feature selection problem, optimization, metaheuristics, classifier, evaluation metrics, objective function, systematic literature review

## Abstract

Feature selection is becoming a relevant problem within the field of machine learning. The feature selection problem focuses on the selection of the small, necessary, and sufficient subset of features that represent the general set of features, eliminating redundant and irrelevant information. Given the importance of the topic, in recent years there has been a boom in the study of the problem, generating a large number of related investigations. Given this, this work analyzes 161 articles published between 2019 and 2023 (20 April 2023), emphasizing the formulation of the problem and performance measures, and proposing classifications for the objective functions and evaluation metrics. Furthermore, an in-depth description and analysis of metaheuristics, benchmark datasets, and practical real-world applications are presented. Finally, in light of recent advances, this review paper provides future research opportunities.

## 1. Introduction

Recently, the interest in artificial intelligence, particularly machine learning, has increased. The great success of this technology is due to the extensive computing capacity now available and the vast volumes of existing data. The high level of available data has allowed machine learning algorithms to become increasingly complex and they can be nourished from different sources. This can be a great benefit, but it can also be a problem since the data may be redundant and irrelevant, causing learning errors [1]. In this context, a set of features that describe the problem studied is defined based on existing data. When the datasets used are large, extensive sets of features are generated, and eliminating irrelevant information is of the utmost importance. The feature selection problem consists of finding a subset of features that represents the original dataset with the aim of eliminating irrelevant and redundant information within the dataset to improve the performance of a classification algorithm [2]. This problem is complex since the search space is defined as $2^{n}$, where *n* corresponds to the number of features that make up the dataset [3].

**Figure 1:**
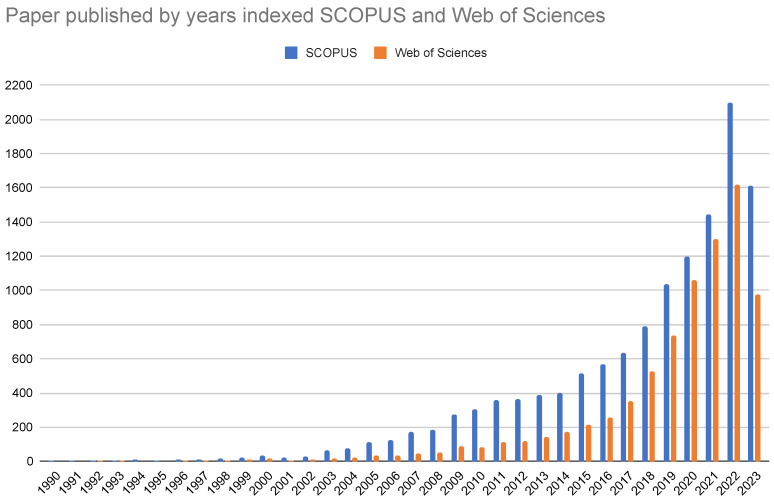
Papers published by year indexed Scopus and Web of Science.

To initially analyze the information related to the feature selection problem, we performed a simple search on Web of Science and Scopus using the terms “Feature Selection” and “Optimization”, obtaining 8016 and 12,908 research papers, respectively. Figure 1 shows the number of publications per year, evidencing the increase in the study of the problem, especially in recent years.

Given the extensive number of investigations on the topic, many methods for solving the problem have emerged. Following the extensive related literature [3,4,5,6,7,8,9,10,11,12,13,14], the solution methods for the feature selection problem can be classified as shown below:

- Filter methods identify the optimal set of features by focusing on the specificities of the problem within the dataset without considering the classification algorithm to be used. These methods use statistical analysis to explore the connection between each input and target variable, assigning a relevance value to each feature. They stand out for their speed and computational efficiency. Examples of these methods include the correlation coefficient, the chi-squared test, and the Fisher score.
- Wrapper methods approach the feature selection iteratively, continuously adjusting the subset of features based on the training phase of the machine learning model. These methods offer a set of features ideally suited to the needs of the model and often performance improvement. Among its most well-known categories are forward selection, backward elimination, exhaustive selection, and metaheuristics.
- Embedded methods were introduced to overcome the difficulties filter and wrapper methods face. The purpose is to obtain quick results and with greater accuracy. Examples include lasso regression, decision trees, and random forest algorithms.

Wrapper methods are computationally more expensive than filter methods; however, the former delivers better results. Metaheuristics stand out within the wrapper methods.

Metaheuristics are a general purpose algorithm that with few modifications can solve different optimization problems. They are algorithms with stochastic behavior whose optimization process is performed by balancing the exploration of the search space and the exploitation of promising regions [15], and these features make metaheuristics deliver high-quality results in a reasonable time. In the literature, we can find different metaheuristics that are inspired by physical phenomena, evolutionary theory, and animal social behavior [16]. The great variety of existing metaheuristics is due to the no free lunch theorem, which indicates that there is no optimization algorithm capable of solving all existing optimization problems [17,18,19]. In other words, this theorem motivates us to continue innovating and experimenting with new metaheuristics, making hybridizations, and developing modifications to metaheuristics. In this sense, Becerra-Rozas et al. [20] reviewed the literature related to the binarization of continuous metaheuristics to solve combinatorial problems, finding that the feature selection problem is highly studied and providing inspiration to continue exploring the field of metaheuristic binarization. In addition, in the related literature, several literature reviews address the use of metaheuristics to solve the problem. These works mainly focus on aspects related to the objective function, evaluation metrics, metaheuristics, classifiers, benchmarks, and real-world applications. Table 1 shows a summary of the contributions found since 2015 in the literature ordered by year of publication. The “✓” indicates that the literature review analyzed includes the field seen in the column. The last row indicates the contributions we made in our literature review.

In most literature reviews, the authors do not detail the objective functions pursued in the corresponding compiled works. Similarly, the evaluation metrics used by the authors are only defined in detail in [3,13]. On the other hand, the metaheuristics used to solve the problem are the common aspects studied in the mentioned reviews. The authors address the binarization of metaheuristics in [4,5,6,10]. For example, in [5], the authors detail whether the metaheuristics were binarized or modified with chaotic maps. In addition, different classifications based on behavior, hybridization, the main modifications carried out, and real-world applications, among others, are presented in [4,5,6,8,9,11,13]. Also, in investigations such as [3,7,8,9,12,13], the authors indicate the contribution made by the metaheuristic to the field of feature selection. The works that address the different classifiers commonly define each classifier used or at least the most common ones and, for example, in [6], the authors additionally indicate the statistical tests used to validate the obtained results. Regarding the datasets used, classifications are also presented. For example, in [7], the authors classify the datasets used according to size, field, and number of classes. Finally, applications in the real world are also studied; these span across various fields, including, but not limited to, healthcare [21], cybersecurity [22], environmental monitoring [23], and text classification.

As a result of the analysis, we found several aspects not addressed in detail by the existing reviews. This study conducts a systematic literature review presenting a comprehensive taxonomy of objective functions, categorized into single-objective and multi-objective functions. Similarly, a classification is proposed for metrics based on four categories: classifier, metaheuristics, features, and statistical test. In addition, the metaheuristics used to solve the problem are analyzed in depth, emphasizing the implementation details and hybridization. Also, regarding benchmarks and real-world applications, a thorough categorization of repositories is presented, providing standardized, pertinent dataset information.

Based on this, the contributions of this research are the following:

- An updated review of the literature analyzing and discussing objective functions proposed for the feature selection problem as well as metrics, classifiers, and metaheuristics used to solve the problem and benchmarks or real-world applications to validate the results obtained;
- A detailed classification of the objective functions and evaluation metrics provides a better understanding of the status of several aspects.
- A deep analysis of the metaheuristics used by researchers, following different criteria.

The remainder of this document is structured as follows. In Section 2, the applied methodology is presented, also detailing the research questions. In Section 3, a bibliometric analysis of the selected research papers is presented. The research questions proposed in Section 2 are answered in Section 4. Finally, in Section 5, the conclusions of the research and some lines of future work are presented.

## 2. Methodology

The methodology is crucial in ensuring a robust and comprehensive research analysis. This research was conducted following the systematic literature review (SLR) framework [24]. In an SLR, one of the main steps is the definition of the research questions. These questions serve as a compass, directing our exploration and analysis in the area. In this work, the research questions were defined as follows:

- RQ1. How is the objective function of the feature selection problem formulated?
- RQ2. What metrics are used to analyze the performance of the feature selection problem?
- RQ3. What machine learning techniques have been used to calculate fitness in the feature selection problem?
- RQ4. What metaheuristics have been used to solve the feature selection problem?
- RQ5. Which datasets are commonly used as benchmarks, and which are derived from real-world applications?

For the literature review search process, we used six databases well known to the scientific community: Scopus, Web of Science, IEEE Xplore, ScienceDirect by Elsevier, Wiley, and SpringerLink. Our date range was defined between 2019 and 2023 (20 April 2023), and our initial keyword was “Feature Selection”, focusing primarily on titles. Table 2 shows the information regarding the search process for each database. The database is shown in column 1. Columns 2–3 show the query performed and the number of investigations obtained in each database. Owing to the differences in the search and filtering capabilities of these databases, some manual processes were required after obtaining the search results. This involved a refinement process, applying manual year filtering in the case of IEEE Xplore and SpringerLink, and expanding our search criteria.

**Figure 2:**
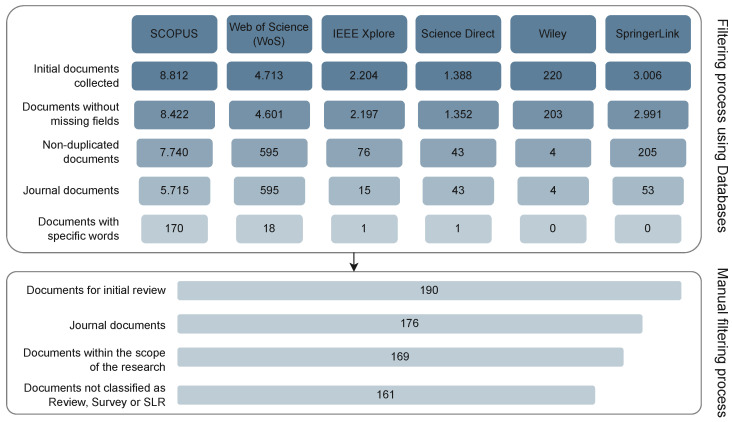
Flowchart of filtering process for the systematic literature review.

Our process of refining the information obtained was based on two main phases, the first making use of the tools provided by the databases, using as inclusion criteria manuscripts that (a) present the title, doi, and abstract, (b) are not duplicates, (c) are published in journals (not in conferences or book chapters), and (d) containing specific words in the abstract, in this case, the phrase “Feature selection problem”. Subsequently, we carried out a manual filtering process on the 190 papers obtained where we verified the previous inclusion criteria as well as manuscripts (e) written in English, (f) within the scope of this research, and (g) not classified as a survey, review, or SLR. Note that this research’s scope refers to manuscripts that use a metaheuristic and present metrics to measure the performance of the proposals and/or the classifier used and/or the optimized objective function and/or the reference datasets/real-world data used. After this process, the final number of manuscripts analyzed in this literature review was 161. Figure 2 shows the process of filtering the collected literature.

## 3. Bibliometric Analysis

To perform a bibliometric analysis, the keywords, year of publication, journal, number of citations, authors, and country of the institution represented by each author were extracted from the selected articles. We used Biblioshiny by [25], a Bibliometrix application developed in R that is open access.

**Figure 3:**
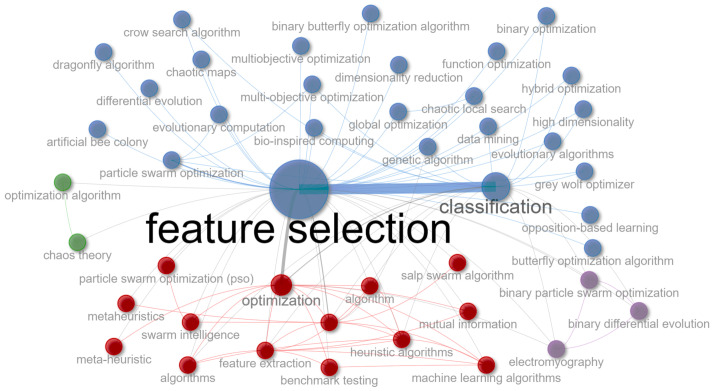
Network map of the keywords found.

Figure 3 shows a network graph plotting the keywords found in the collected papers. Each node symbolizes a keyword, and the size of each node reflects the frequency of occurrence of the corresponding keyword. In the center of this graph are two central nodes, “feature selection” and “classification”, linked together, which means there is a strong correlation between these topics. Additionally, the graph is organized into four main groups, indicated by a different color: blue, red, green, and purple. This codification of colors is an effective network graph technique for visually differentiating groups of nodes that often interact or are related. These clusters suggest a typical grouping pattern of these keywords, revealing underlying connections and thematic consistencies within the research field.

**Figure 4:**
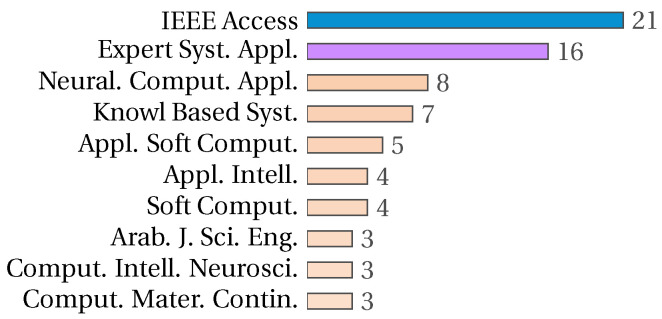
Research by journal.

**Figure 5:**
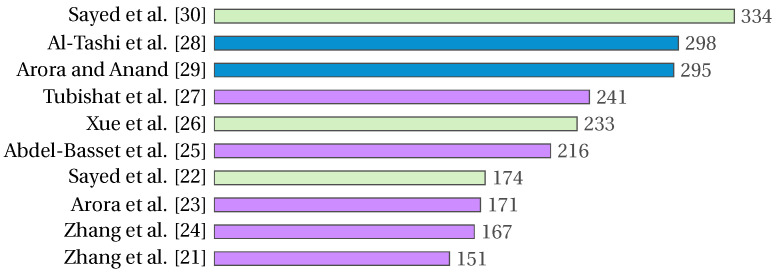
Citations by author [21,22,23,24,25,26,27,28,29,30].

IEEE Access is the journal with the most research related to the feature selection problem, followed by Expert System with Applications. These data are in accordance with the most cited papers on the feature selection problem. Figure 4 shows the ten journals with the most research on the feature selection problem. Figure 5 shows the top ten cited papers, with the second and third places corresponding to articles published in IEEE Access, and five of the ten most cited papers having been published in Expert System with Applications. In both figures, blue and violet represent the information relating to IEEE Access and Expert System with Applications, respectively.

**Figure 6:**
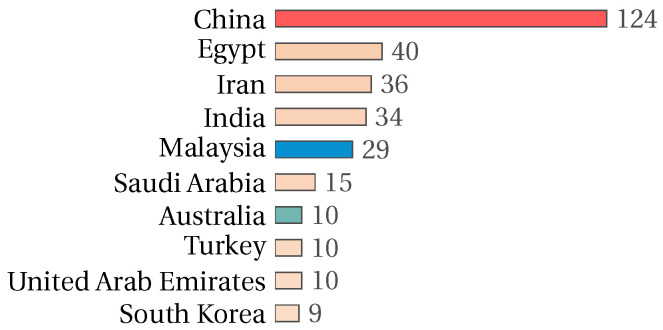
Research by country.

**Figure 7:**
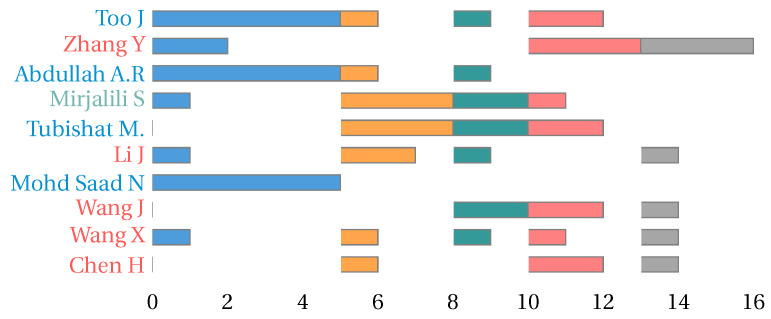
Research by authors by years.

On the other hand, when analyzing the contributions by country, we found that China presents the most significant number of published papers related to the feature selection problem. This is in accordance with the contributions by authors, where five of the ten most active authors represent China, four represent Malaysia, and one represents Australia. Note that the three countries represented by the ten most contributing authors in the area are among the ten countries that present the most significant number of publications. Figure 6 shows the number of investigations on the feature selection problem for the ten most prominent countries. Figure 7 shows the annual contribution of the ten most representative authors in the area. The colors blue, orange, green, red, and gray in the bars represent the papers published from 2019 to 2023, respectively. The color of the name of the author is related to the country represented.

## 4. Discussion

### 4.1. How Is the Objective Function of the Feature Selection Problem Formulated?

**Figure 8:**
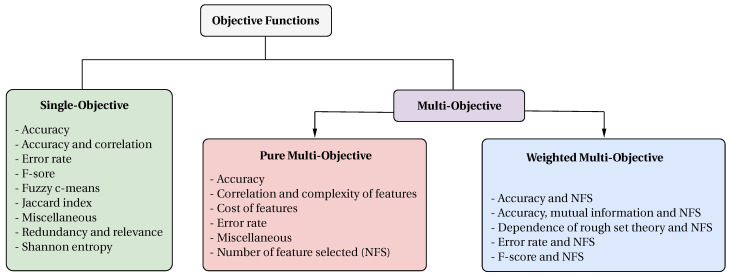
Taxonomy of objective functions.

Optimization problems are composed of an objective function subject to constraints. The objective functions can be classified into two main categories: single-objective, focused on optimizing only one objective; and multi-objective, focused on optimizing several objective functions at the same time. There are two ways of representing multi-objective optimization problems: (1) a pure multi-objective function, and (2) a weighted multi-objective function. Figure 8 summarizes the classification of the objective functions found in the papers collected.

**Figure 9:**
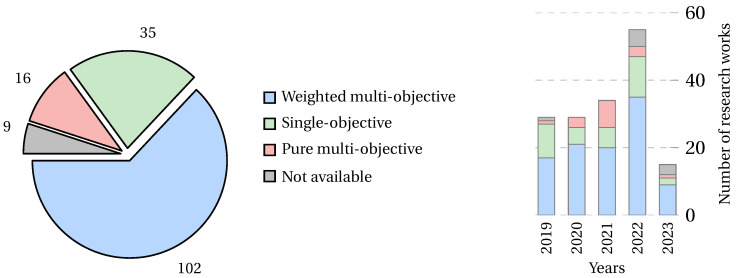
Objective functions by category.

Multi-objective functions are pursued in 73% of the collected research, with the weighted multi-objective function being the most used objective function classification in the related literature. This trend occurs in general and over the years. Figure 9 shows the number of papers that have pursued different objective function classifications in general (left) and their trend over the years (right).

#### 4.1.1. Single-Objective Functions

Single-objective functions are focused on optimizing only one objective function subject to constraints. Mathematically, single-objective optimization problems are modeled as follows [26]:

(1) minormaxf(X)

Subject to

(2) $$\begin{array}{c} g_{i}\left(X\right)<0\;i=1,2,\ldots ,N_{ieq}\\ h_{i}\left(X\right)=0\;i=1,2,\ldots ,N_{eq} \end{array}$$

where f(X) represents the objective function, *X* corresponds to the solution vector composed of the decision variables, and $g_{i}\left(X\right)$ and $h_{i}\left(X\right)$ are the inequality and equality constraints, respectively. Within this category, eight different functions were detected:

(**a**) **Accuracy**: Defined in detail in Section 4.2.1 and mathematically in Equation (22). This objective function was pursued in [21,27,28,29,30,31,32,33,34,35,36,37,38,39,40,41].

(**b**) **Error rate**: Defined in detail in Section 4.2.1 and mathematically in Equation (26). This objective function was pursued in [42,43,44,45,46,47,48,49,50,51].

(**c**) **Fuzzy c-means (FCM)**: Clustering algorithm which returns a cost function used to calculate the performance of the metaheuristic [52]. This objective was pursued in [53].

(**d**) **Redundancy and relevance**: Redundancy has been used to quantify the similarity level between selected features. Relevance represents the relevance between features and categorical variables reflecting the recognition ability of the selected features. We found two papers that pursued relevance and redundancy. In [54], the authors use these metrics to calculate the objective function and relate them by subtraction, as follows:

(3) F(X)=Relevance−Redundancy

On the other hand, in [55], the authors relate these metrics by means of a division, as shown below:

(4) $$F\left(X\right)=\frac{Redundancy}{Relevance}$$

(**e**) **Accuracy and correlation**: In [56], the authors present an objective function that relates the correlation between the selected features without the presence of class labels and the accuracy. This objective function was pursued in [56] and mathematically is defined as follows:

(5) $$F\left(X\right)=\frac{A+(1-M)}{2}$$

where *A* is the accuracy and *M* is the computed correlation.

(**f**) **Shannon entropy**: Measures the amount of information in a distribution. If a distribution has a high entropy value, it contains more information. The authors of [57] used this information as an objective function, defining it mathematically as follows:

(6) $$F\left(X\right)=-\sum_{i=1}^{n}p\left(x_{i}\right)log_{2}p\left(x_{i}\right)$$

where *n* corresponds to the number of features and $p(x_{i})$ is the probability of occurrence of a feature.

(**g**) **Humming loss**: Defined in detail in Section 4.2.1 and mathematically in Equation (23). This objective function was pursued in [22].

(**h**) **Jaccard index**: Measures the similarity and overlap between two sets. It is often used in data analysis, information retrieval, and text mining. This objective function was pursued in [58] and mathematically is defined as follows:

(7) $$F\left(X\right)=\frac{TP}{TP+FP+FN}$$

where TP (true positive) is the number of positive instances correctly classified; FP (false positive) is the number of negative instances wrongly classified as positive; and FN (false negative) is the number of positive instances wrongly classified as negative.

(**i**) **Miscellaneous**: Finally, we detected two objective functions which the authors explain in greater detail in the respective papers; see [59,60].

**Figure 10:**
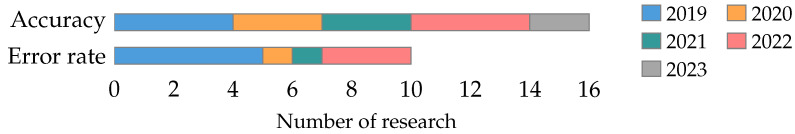
Single-objective functions by year.

Accuracy is the most studied single-objective function in the collected literature, present in 10% of the research, and maintains a stable behavior in terms of research per year. Figure 10 shows the number of papers by year that have pursued the two most studied single-objective functions in the collected literature.

#### 4.1.2. Pure Multi-Objective Functions

Pure multi-objective functions are focused on independent optimization of each objective function. Thus, Pareto dominance is used to determine the best solution. Mathematically, multi-objective optimization problems are modeled as follows [61]:

(8) $$\min\;or\;\max\;f_{1}\left(X\right),f_{2}\left(X\right),\ldots ,f_{m}\left(X\right)$$

Subject to

(9) $$\begin{array}{c} g_{i}\left(X\right)<0\;i=1,2,\ldots ,N_{ieq}\\ h_{i}\left(X\right)=0\;i=1,2,\ldots ,N_{eq} \end{array}$$

where $f_{1}\left(X\right),f_{2}\left(X\right),\ldots ,f_{m}\left(X\right)$ represents the *m* objective functions to be optimized, *X* corresponds to the solution vector composed of the decision variables, and $g_{i}\left(X\right)$ and $h_{i}\left(X\right)$ are the inequality and equality constraints, respectively. Within the category of pure multi-objective functions, six different functions were detected:

(**a**) **Error rate**: Defined in Section 4.2.4 and mathematically in Equation (26). This objective function has been used in [62,63,64,65,66,67,68,69,70,71,72,73].

(**b**) **Number of features selected (NFS)**: One of the essential aspects when solving the feature selection problem is to increase the performance of the classifiers to the smallest number of features possible. Given this, the number of selected features is an important objective, pursued in [63,64,65,66,67,68,69,70,71,72,73,74].

(**c**) **Cost of features**: In [62], the authors incorporate the costs associated with features to the feature selection problem, minimizing the costs associated with the features and the error rate of the classification algorithms.

(**d**) **Accuracy**: Defined in Section 4.2.3 and mathematically in Equation (22). This objective function was pursued in [74].

(**e**) **Correlation and complexity of features**: In [63], the authors propose four different metrics to build the objective function, and join to the error rate and the correlation and complexity of the features.

(**f**) **Miscellaneous**: In [75], the authors use six different metrics to build the objective function, defined in Section 4.2.1 and named as follows:

- $F_{1}\left(S\right)$ = Number of features selected;
- $F_{2}\left(S\right)$ = Accuracy;
- $F_{3}\left(S\right)$ = Relevance;
- $F_{4}\left(S\right)$ = Redundancy;
- $F_{5}\left(S\right)$ = Interclass Distance;
- $F_{6}\left(S\right)$ = Intraclass Distance.

Thus, the objective function is defined as follows:

(10) $$\min\;F\left(X\right)=F_{1}\left(S\right),-F_{2}\left(S\right),-F_{3}\left(S\right),F_{4}\left(S\right),-F_{5}\left(S\right),F_{6}\left(S\right)$$

**Figure 11:**
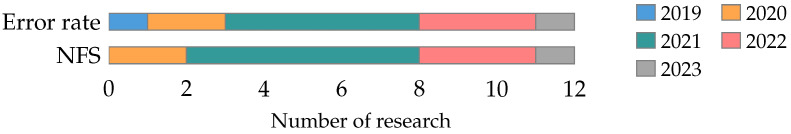
Pure multi-objective functions by year.

Error rate and NFS are the most studied pure multi-objective functions in the collected literature, each one is studied in 7% of the research and shows a considerable increase in 2021. Figure 11 shows the number of papers by year that have pursued the two most studied pure multi-objective functions in the collected literature.

#### 4.1.3. Weighted Multi-Objective Functions

In general, metaheuristics are designed to solve single-objective optimization problems, and adapting them to multi-objective optimization problems is very costly both computationally and in development time. In [76], the authors present a way to translate a multi-objective optimization problem into a single-objective optimization problem. This procedure is a weighted sum of all the objective functions, and mathematically it is defined as follows:

(11) $$\min\;or\;\max\;f\left(X\right)=w_{1}f_{1}+w_{2}f_{2}+\ldots +w_{m}f_{m}$$

Subject to

(12) $$\begin{array}{c} w_{i}\geq 0\;\forall i=1,2,\ldots ,m\\ w_{1}+w_{2}+\ldots +w_{m}=1 \end{array}$$

where $w_{1},w_{2},\ldots ,w_{m}$ are non-negative weights for *m* objective functions. Within the category of weighted multi-objective functions, five different ones were detected:

(**a**) **Error rate and number of features selected (error rate and NFS)**: Within the feature selection problem, it is essential to improve the performance of the classifier and reduce the number of features. Given this, a weighted multi-objective function that relates these two terms was proposed as follows:

(13) $$F\left(X\right)=\alpha \cdot Error\;Rate+\beta \cdot \frac{S}{F}$$

where *S* and *F* correspond to the number of features selected and the total number of features of the dataset, and α and β assign the importance of the error rate and the number of features selected. α and β∈[0,1], and there is no consensus on the values of these parameters. This objective function was pursued in [77,78,79,80,81,82,83,84,85,86,87,88,89,90,91,92,93,94,95,96,97,98,99,100,101,102,103,104,105,106,107,108,109,110,111,112,113,114,115,116,117,118,119,120,121,122,123,124,125,126,127,128,129,130,131,132,133,134,135,136,137,138,139,140,141,142,143,144,145,146,147,148,149,150,151,152,153,154,155,156,157,158,159,160,161].

(**b**,**c**) **Accuracy and number of features selected (accuracy and NFS)**: Objective function similar to error rate and NFS. The difference is that the accuracy is the metric of the classification technique. In the literature, two objective functions that associate accuracy with the number of features selected were detected.

The first version found is defined as follows:

(14) $$F\left(X\right)=Accuracy+\alpha \cdot \left(1-\frac{S}{F}\right)$$

where *S* and *F* correspond to the number of features selected and the total number of features of the dataset, and α assigns the importance of the number of features selected. α∈[0,1] and there is no consensus on the values of this parameter. This objective function was pursued in [130,162,163,164,165].

The second version found is defined as follows:

(15) $$F\left(X\right)=\alpha \cdot Accuracy+\beta \cdot \left(\frac{F-S}{F}\right)$$

where *S* and *F* correspond to the number of features selected and the total number of features of the dataset, and α and β assign the importance of the accuracy and the number of features selected. α and β∈[0,1], and there is no consensus on the values of these parameters. This objective function was pursued in [166,167,168,169,170,171,172,173,174].

(**d**) **F-score and number of features selected (F-score and NFS)**: This objective function has only been studied in [175] and mathematically is defined as follows:

(16) $$\min\;F\left(X\right)=w_{1}z_{1}+w_{2}z_{2}+w_{3}z_{3}$$

where $w_{1}+w_{2}+w_{3}=1$ and the authors determine that $w_{1}=0.5$, $w_{2}=0.25$, and $w_{3}=0.25$. $z_{1}$, $z_{2}$, and $z_{3}$ are defined as follows:

(17) $$\min\;z_{1}=1-F\text{-}\mathrm{score}$$

(18) $$\min\;z_{2}=\frac{\left|S\right|}{T}$$

(19) $$\min\;z_{3}=\frac{\max\{t|x_{t}\in S\}}{T}$$

where $z_{1}$ aims to maximize the F-score, $z_{2}$ seeks to minimize the number of features selected per unit of time, and $z_{3}$ pursues the minimize the last feature to be selected.

(**e**) **Accuracy, mutual information, and number of features selected**: This objective function has only been studied in [176] and mathematically, is defined as follows:

(20) $$F\left(X\right)=\alpha \cdot \mathrm{Accuracy}+\beta \cdot \left(\frac{\left|F-S\right|}{F}\right)+\gamma \cdot \mathrm{Mean}\left(I\left(X_{k};Y\right)\right)$$

where |S| is the number of selected features, α·Accuracy, $\beta \left(\frac{\left|F-S\right|}{F}\right)$ and $\delta \cdot \mathrm{Mean}(I\left(X_{k};Y\right))$ are considered for increasing the classification accuracy, reducing the number of selected features, and increasing the mean of the mutual information, respectively. α, β, and δ∈[0,1] and the sum equals 1.

(**f**) **Dependence of rough set theory and number of features selected**: This objective function has only been studied in [177] and mathematically is defined as follows:

(21) $$F\left(X\right)=\alpha \cdot dep\left(X\right)+\beta \cdot \frac{1}{S}$$

where *X* is the feature subset found. Fitness is calculated based on the dependency measure of rough set theory dep(X), and *S* is the length of the feature subset $\mathrm{size}(x_{i})$. α∈[0,1] controls the relative weight of the dependency value and feature subset length, and β is (1−α).

**Figure 12:**
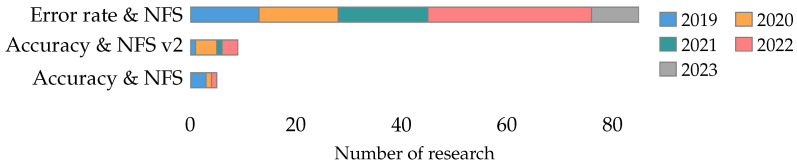
Weighted multi-objective functions by year.

The objective functions found in this classification combine the number of features selected. In this sense, the most studied combination in the literature is error rate and NFS, present in 53% of the research. Figure 12 shows the number of papers per year that have pursued the three most studied weighted multi-objective functions in the collected literature.

### 4.2. What Metrics Are Used to Analyze the Performance of the Feature Selection Problem?

**Figure 13:**
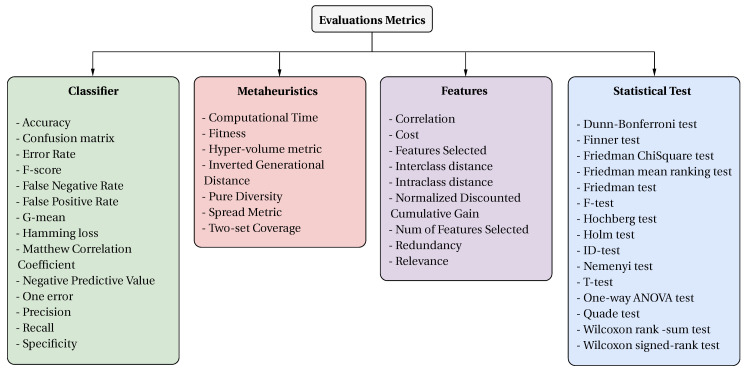
Taxonomy of evaluation metrics.

To facilitate the understanding of the different metrics found in the literature, the collected papers were grouped into four categories according to the metrics used. Figure 13 shows the proposed classification for the metrics found.

**Figure 14:**
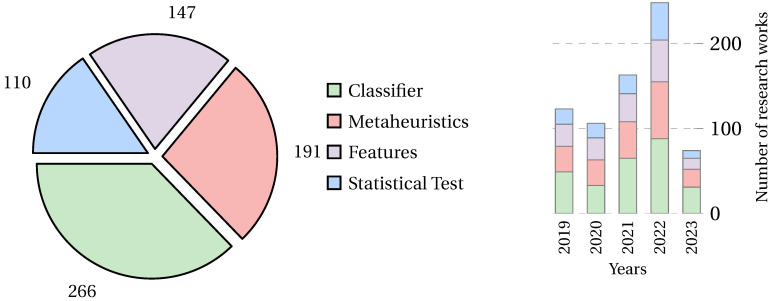
Evaluation metrics by category.

Classifiers are the most used metric in the collected literature, followed by metaheuristic metrics, with statistical tests being the least used. Figure 14 shows the number of papers that have used the different metric classifications in general (left) and their trends over the years (right).

#### 4.2.1. Classifier Metrics

The classifier metrics measure the performance of the machine learning technique using the subset of features obtained from solving the feature selection problem. In the literature, we detected 14 metrics of the classifier.

(**a**) **Accuracy**: Measures how well a classification algorithm correctly predicts the classes of the datasets. Accuracy is calculated as the ratio of correctly predicted cases to the total number of cases in the dataset. This metric has been used in [21,22,27,28,29,30,31,32,33,34,35,36,37,38,39,40,41,42,43,44,45,46,47,48,50,51,53,54,55,56,57,58,59,60,65,69,70,72,74,75,77,78,80,81,82,83,84,85,86,87,88,89,90,91,92,94,95,96,97,98,99,101,102,103,104,105,106,107,108,109,110,111,112,113,114,115,116,117,118,119,121,124,125,126,127,128,129,130,131,132,133,135,136,137,138,139,141,142,143,144,145,146,147,148,149,150,151,152,153,154,156,158,159,160,161,162,164,165,166,167,168,169,170,171,172,173,174,176,178,179,180,181,182,183,184,185,186] and mathematically is defined as follows:

(22) $$Accuracy=\frac{\mathrm{TP}+\mathrm{TN}}{\mathrm{TP}+\mathrm{TN}+\mathrm{FP}+\mathrm{FN}}$$

where TP (true positive) is the number of positive instances that are correctly classified, FP (false positive) is the number of negative instances that are wrongly classified as positive, TN (true negative) is the number of negative instances that are correctly classified, and FN (false negative) is the number of positive instances that are wrongly classified as negative.

(**b**) **F-score or f1-score or f-measure**: Used when dealing with imbalanced datasets. It combines precision and recall into a single value and is particularly useful for balancing these metrics. This metric has been used in [22,33,35,39,45,48,51,55,59,60,83,92,98,101,117,132,141,149,155,162,164,167,171,176,177,178,182,184,185] and mathematically is defined as follows:

(23) $$f-score=\frac{2\cdot Precision\cdot Recall}{Precision+Recall}$$

where recall and precision are defined in Equation (24) and Equation (25), respectively.

(**c**) **Recall or sensitivity or true positive rate**: Ratio of true positive predictions to the total number of actual positive instances in the dataset. Measures the model’s ability to correctly identify all positive instances. This metric has been used in [22,33,35,55,56,60,70,85,96,98,101,102,106,117,129,132,133,141,149,162,164,171,176,177,178,185] and mathematically is defined as follows:

(24) $$Recall=\frac{\mathrm{TP}}{\mathrm{TP}+\mathrm{FN}}$$

(**d**) **Precision or positive predictive value**: Ratio of true positive predictions to the total number of positive predictions made by the model. Measures the accuracy of the model when it predicts a positive class. This metric has been used in [22,33,35,45,48,51,55,59,74,83,98,101,106,117,129,132,141,149,164,171,176,177,178,185] and mathematically is defined as follows:

(25) $$Precision=\frac{\mathrm{TP}}{\mathrm{TP}+\mathrm{FP}}$$

(**e**) **Error rate**: Sometimes referred to as the misclassification rate, it is an essential complement to accuracy. Measures the overall accuracy of a model in terms of the proportion of misclassified instances in a dataset. This metric has been used in [46,49,59,62,64,65,66,67,68,70,71,93,120,122,131,133,134,140,150,179] and mathematically is defined as follows:

(26) $$Error\;rate=\frac{\mathrm{FP}+\mathrm{FN}}{\mathrm{FP}+\mathrm{FN}+\mathrm{TP}+\mathrm{TN}}\;or\;1-Accuracy$$

(**f**) **Specificity or true negative rate**: Evaluates the ability of the model to correctly identify negative instances (true negatives) out of all the actual negative instances in a dataset. It is essential when the cost of misclassifying a negative instance as positive is high or when seeking to emphasize the ability of the model to classify negative cases correctly. This metric has been used in [33,44,70,85,96,102,129,133,162,176,185] and mathematically is defined as follows:

(27) $$Specificity=\frac{\mathrm{TN}}{\mathrm{TN}+\mathrm{FP}}$$

(**g**) **Matthew correlation coefficient (MCC)**: Assesses the quality of binary and multiclass classifications, especially when dealing with imbalanced datasets. MCC provides a balanced measure of the ability of the model to discriminate between positive and negative instances. This metric has been used in [45,51,181] and mathematically is defined as follows:

(28) $$\mathrm{MCC}=\frac{\mathrm{TN}\cdot \mathrm{TP}-\mathrm{FN}\cdot \mathrm{FP}}{\sqrt{\left(\mathrm{TP}+\mathrm{FP})\cdot (\mathrm{TP}+\mathrm{FN})\cdot (\mathrm{TN}+\mathrm{FP})\cdot (\mathrm{TN}+\mathrm{FN}\right)}}$$

MCC ranges from −1 to 1, where an MCC = 1 indicates a perfect classification, an MCC = 0 suggests random classification, and an MCC = −1 indicates a complete disagreement between predictions and actual values.

(**h**) **False positive rate (FPR)**: Measures the rate at which the model incorrectly classifies negative instances as positive. It is an important metric where the cost of false positives is significant. This metric has been used in [33,58,98] and mathematically is defined as follows:

(29) $$FPR=\frac{\mathrm{FP}}{\mathrm{FP}+\mathrm{TN}}$$

(**i**) **False negative rate (FNR)**: Measures the rate at which the model incorrectly classifies positive instances as negative. It is essential when failing to detect positive instances has significant consequences. This metric has been used in [33,58] and mathematically is defined as follows:

(30) $$FNR=\frac{\mathrm{FN}}{\mathrm{TP}+\mathrm{FN}}$$

(**j**) **Hamming loss**: Evaluates the performance of multi-label classification problems. In multi-label classification, each instance can be associated with multiple class labels, and the goal is to predict all the correct labels for each instance. The Hamming loss quantifies how well the model performs in terms of correctly predicting all the labels for each instance. This metric has been used in [41,63] and mathematically is defined as follows:

(31) $$Hamming\;Loss=\frac{1}{p}\sum_{i=1}^{p}\frac{1}{q}|h\left(x_{i}\right)△Y_{i}|$$

where *p* and *q* indicate the number of samples and labels. $h(x_{i})$ represents the result of classification for the i-th sample and $Y_{i}$ shows the actual labels of the corresponding sample. $h(x_{i})$ and $Y_{i}$ are binary vectors in which 1’s indicate the class labels to which the instances belong. △ represents the Hamming distance between $h(x_{i})$ and $Y_{i}$.

(**k**) **One error**: Evaluates how often the model makes exactly one error when predicting the set of labels for an instance. This metric has been used in [41,63] and mathematically this metric is defined as follows:

(32) $$One\;Error=\frac{1}{\left|T\right|}\sum_{i=1}^{\left|T\right|}\left[\arg\max_{l_{k}\in L}g_{k}\left(w_{i}\right)\notin \lambda_{i}\right]$$

Let $T=\{\left(w_{i},\lambda_{i}\right)|1\leq i\leq |T|\}$ be a given test set, where $\lambda_{i}\subseteq L$ is a correct label subset that is associated with a pattern $w_{i}$. Here, *L* represents the set of all possible labels in the multi-label classification task. Given a test pattern $w_{i}$ and a multi-label classifier estimate a predicted label set $Y_{i}\subseteq L$. Specifically, a series of functions $\left\{g_{1},g_{2},\ldots ,g_{\left|L\right|}\right\}$ is induced from the training patterns. Next, each function $g_{k}$ determines the class membership of $l_{k}$ with respect to each pattern, i.e., $Y_{i}=\{l_{k}|g_{k}\left(w_{i}\right)>\theta ,1\leq k\leq \left|L|\right\}$, where θ is a predetermined threshold, such as 0.5.

(**l**) **Confusion matrix**: Provides a comprehensive and detailed model performance summary. It is particularly useful for evaluating the quality of predictions, understanding the types of errors a model makes, and assessing its strengths and weaknesses. This metric has been used in [149,185] and is defined as a square matrix where each row represents the actual class labels, and each column represents the predicted class labels. Table 3 shows the confusion matrix of a binary classification.

(**m**) **G-mean or geometric mean**: Calculated as the geometric mean of sensitivity and specificity. Sensitivity measures the model’s ability to correctly identify positive instances, while specificity measures its ability to identify negative instances correctly. This metric has been used in [70] and mathematically is defined as follows:

(33) $$G-mean=\sqrt{Sensitivity\cdot Specificity}$$

where sensitivity and specificity are defined in Equations (24) and (27).

(**n**) **Negative predictive value (NPV)**: Used mainly in medical and diagnostic applications, it assesses the ability of a model to identify correctly negative instances among those it predicts as negative. This metric has been used in [162] and mathematically is defined as follows:

(34) $$NPV=\frac{TN}{TN+FN}$$

**Figure 15:**
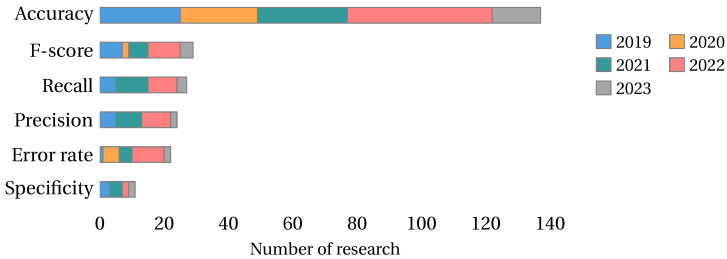
Classifier metrics by year.

Accuracy is the most used classifier metric in the literature, presented in 85% of the collected research. On the other hand, the rest of the classifier metrics are studied in less than 25% of the research. Figure 15 shows the classifier metrics studied by year.

#### 4.2.2. Metaheuristic Metrics

The metaheuristic metrics measure the performance of the metaheuristic when solving the feature selection problem. Within this classification, seven different metaheuristic metrics were detected.

(**a**) **Fitness**: Corresponds to the evaluation of the objective function of the solution obtained. The authors report the best and worst solutions obtained, the average of the different executions performed, and their standard deviation. This metric has been used in [21,31,33,46,48,50,53,54,57,60,78,79,80,81,82,84,85,87,88,89,90,91,92,93,94,95,96,97,98,99,101,102,103,105,106,107,108,110,112,114,115,118,119,120,121,122,123,124,125,126,127,128,129,130,131,132,133,134,135,136,137,138,139,140,142,143,144,145,146,147,148,149,151,152,153,154,155,157,158,159,160,161,163,164,165,166,167,172,175,177,179,183,185]

(**b**) **Computational time (CT)**: Corresponds to the computational times used by the algorithms to solve the feature selection problem. It is important to note that the authors report the CPU, operating system, RAM capacity, and programming language used in the experimental environment. The authors report the times in seconds or minutes and present the average obtained in the different executions performed as their standard deviation. This metric has been used in [21,22,34,36,38,39,44,48,49,51,53,55,65,67,69,71,72,75,77,80,81,82,83,84,85,86,87,89,91,98,105,106,107,108,109,113,114,115,116,119,125,126,128,132,134,138,139,140,142,143,144,145,146,147,148,157,158,160,163,165,166,167,168,169,170,171,172,176,178,179,181,182,184,186]

(**c**) **Hyper-volume (HV)**: Used for pure multi-objective optimization problems and measures the hyper-volume of a region formed by the solution set and a reference point. The reference point usually is the anti-optimal point or “worst possible” point in the objective space. This metric has been used in [62,63,64,65,67,68,69,70,73,75,120,179]

(**d**) **Inverted generational distance (IGD)**: Computes the average Euclidean distance from true Pareto fronts to its closest solution in the population. This metric has been used in [64,65,68,73,75] and mathematically is defined as follows:

(35) $$IGD\left(S\right)=\frac{\sum_{x^{*}\in F^{*}}dist\left(x^{*},S\right)}{|F^{*}|}$$

where *S* is the current Pareto front set, $dist(x^{*},S)$ corresponds to the Euclidean distance between a point $x^{*}\in F^{*}$ and its nearest solution *S*.

(**e**) **Two-set coverage (SC)**: Used for pure multi-objective optimization problems and employed to compare the convergence degree of two algorithms. This metric has been used in [62,63,70] and mathematically is defined as follows:

(36) $$SC\left(A,B\right)=\frac{\left|\{b\in B|\exists a\in A:a\;dominates\;b\}\right|}{\left|B\right|}$$

where *A* and *B* are two Pareto fronts obtained by two algorithms, and SC(A,B) is defined as the percentage of solutions in *B* that are dominated by at least one solution in *A*.

(**f**) **Pure diversity (PD)**: Monitor the diversity of solutions during the optimization process. This metric has been used in [63,65] and mathematically is defined as follows:

(37) $$PD\left(S\right)=\max_{s_{i}\in S}\left\{PD\left(S-s_{i}\right)+d\left(s_{i},S-s_{i}\right)\right\}$$

where *S* is the current Pareto front, and $d(s_{i},S-s_{i})$ denotes the dissimilarity *d* from one solution $S_{i}$ to the population *S*.

(**g**) **Spread**: Measures the extent of spread achieved among the obtained non-dominated solutions. This metric has been used in [65,179] and mathematically is defined as follows:

(38) $$Spread\left(S\right)=\frac{d_{f}+d_{l}+\sum_{i=1}^{N-1}\left|d_{i}-\bar{d}\right|}{d_{f}+d_{l}+\left(N-1\right)\bar{d}}$$

where $d_{i}$ is the Euclidean distance between neighboring solutions on the obtained non-dominated solutions set and $\bar{d}$ is the mean of all $d_{i}$. The parameters $d_{f}$ and $d_{l}$ are the Euclidean distances between the extreme and boundary solutions of the obtained non-dominated set. *N* is the number of non-dominated solutions found so far.

**Figure 16:**
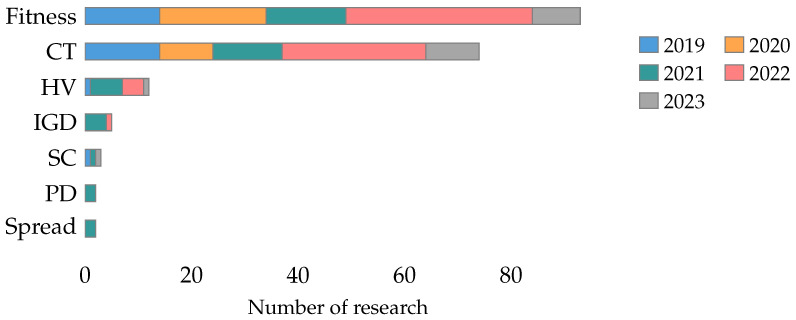
Metaheuristic metrics by year.

Fitness and computational time are the most used metaheuristic metrics in the literature, present in 58% and 46% of the collected papers. The rest of the metaheuristic metrics are studied in less than 8% of the literature. Figure 16 shows the metaheuristic metrics studies by year.

#### 4.2.3. Feature Metrics

Feature metrics measure the attributes of the features that comprise the subset of selected features. Within this classification, nine different feature metrics were detected.

(**a**) **Number of features selected (NFS)**: Corresponds to the number of features that make up the best subset of features. The authors report the average number of features selected in the performed runs and their standard deviation. This metric has been used in [21,27,28,32,34,36,37,38,40,42,44,45,46,47,48,49,50,51,53,54,55,57,58,63,64,65,66,67,68,69,70,71,72,75,77,78,81,82,83,84,85,86,87,88,89,90,91,92,93,94,95,96,97,98,99,101,102,103,104,105,106,107,108,109,110,111,112,113,114,115,116,117,118,119,120,121,122,124,125,126,127,128,129,130,131,132,133,134,135,136,137,138,139,140,142,143,144,145,146,149,151,152,153,155,156,157,158,159,160,161,162,163,164,165,166,167,168,169,170,171,172,173,174,176,177,182,183,184,185,186,187]

(**b**) **Feature selected (FS)**: Corresponds to the identification of the selected characteristics. According to what has been detected in the literature, it is not only enough to indicate how many features were selected, but it is also essential to indicate which were selected. This metric has been used in [29,33,53,58,62,81]

(**c**) **Cost**: Considers the costs associated with the features. This cost can be either the cost of procurement or processing. This metric has been used in [62]

(**d**) **Normalized discounted cumulative gain (NDCG)**: Measures the effectiveness of a ranking algorithm by considering the relevance of the items ranked and their positions in the list. NDCG is an extension of the discounted cumulative gain metric, normalized to provide a score between 0 and 1, making comparing different rankings or recommendation systems easier. This metric has been used in [74]

(**e**) **Correlation**: Used in multi-class classification and seeks to determine the correlation between features and classes. This metric has been used in [63] and mathematically is defined as follows: Given a set of features (F) and a set of labels (L), the correlation $r_{fl}$ between the features f∈F and the label l∈L should be calculated using a predefined measure. The average feature-label correlation is required over all labels and all features. This average is calculated as follows:

(39) $$\bar{r_{FL}}=\frac{1}{F\cdot L}\sum_{f\in F}\sum_{l\in L}r_{fl}$$

(**f**) **Relevance**: Represents the relevance between features and categorical variables and reflects the recognition ability of the selected features. This metric has been used in [75].

(**g**) **Redundancy**: Quantifies the level of similarity between selected features. This metric has been used in [75].

(**h**) **Interclass distance**: Represents the distance between the mean sample of each class and the average of the mean samples of all classes. This metric has been used in [75] and mathematically is calculated as follows:

(40) $$Interclass\;distance=\sum_{i=i}^{L}{\left(m_{i}-\frac{1}{L}\sum_{i=1}^{L}m_{i}\right)}^{2}$$

where *L* is the total number of classes and $m_{i}$ is the average value of all samples with feature *S* in class *i*.

(**i**) **Intraclass distance**: Reflects the cohesion of the same type of samples. It is calculated by the distances between the samples with the selected characteristic and the average of all samples of the same type. This metric has been used in [75] and mathematically is calculated as follows:

(41) $$Intraclass\;distance=\sum_{i=1}^{L}\;\sum_{a_{ij}\in L_{i}}{\left(a_{ij}-m_{i}\right)}^{2}$$

where $a_{ij}$ is the *j*-th sample in class *i*.

**Figure 17:**
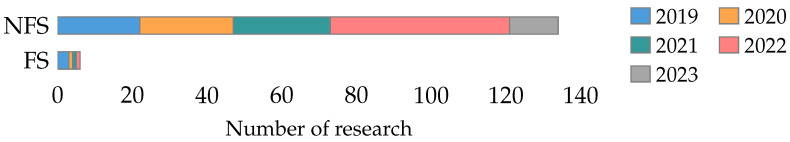
Feature metrics by year.

The number of features selected (NFS) is the most used feature metric in the literature, present in 83% of the collected papers. Features selected (FS) is the other metric used in more than one study. The rest of the feature metrics are studied in only one study. Figure 17 shows the feature metrics studied by year.

#### 4.2.4. Statistical Tests

To demonstrate that one proposal is better than another, improvements must be shown in a particular metric, and a statistical test must be applied. Within this classification, 15 different statistical tests were detected.

(**a**) **Non-parametric statistical test**: Used to make inferences about data when the assumptions of parametric tests are not met. To apply a non-parametric statistical test, (1) the data do not follow a normal distribution; (2) the data are measured on an ordinal or nominal scale rather than on a continuous scale; (3) the assumption of homogeneity of variances are violated; and (4) the sample size is small, making it difficult to rely on the central limit theorem to approximate a normal distribution. In this systematic review, six non-parametric statistical tests were detected. (**i**) **Wilcoxon signed-rank test or Wilcoxon test** [29,36,48,50,59,78,79,85,88,90,91,95,99,108,110,115,119,121,124,125,126,130,131,136,137,138,142,148,151,152,159,165,169,170], (**ii**) **Friedman test** [39,57,59,75,77,94,96,107,108,120,128,132,136,140,142,154,161,179,184], (**iii**) **Wilcoxon rank-sum test or Mann–Whitney U test** [30,38,49,72,82,86,94,97,98,117,129,139,145,157,160,163,164,167,173,176], (**iv**) **Friedman mean ranking test** [112,133,146,179,180], (**v**) **Friedman chi-square test** [128,137] and (**vi**) **Iman–Davenport test** [27].

(**b**) **Parametric statistical test**: Used to make specific assumptions about the underlying probability distribution of the analyzed data. To apply a parametric statistical test, (i) assume that the data follow a specific probability distribution, often the normal distribution; (ii) the data under analysis are continuous; and (iii) homogeneity of variances. In this systematic review, five parametric statistical tests were detected. (**i**) **T-test** [28,39,41,62,70,80,83,105,111,113,127,172,177], (**ii**) **F-test** [27,138], (**iii**) **one-way ANOVA test** [101], (**iv**) **Quade test** [179], and (**v**) **Finner test** [39].

(**c**) **Post hoc analysis**: Statistical procedure after an initial statistical analysis. The term “post hoc” is Latin for “after this.” In the context of statistics, it refers to conducting additional tests or comparisons after the primary analysis to investigate and understand the results further. In this systematic review, four post hoc analyses were detected: (**i**) **Nemenyi test** [27,111,128], (**ii**) **Hochberg test** [57,107,161], (**iii**) **Holm test** [57,107], and (**iv**) **Bonferroni–Dunn test** [41,59].

**Figure 18:**
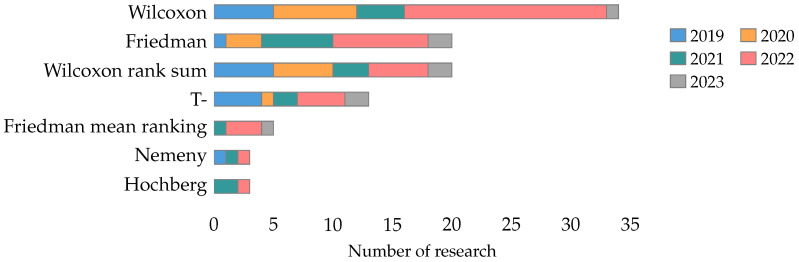
Most used statistical tests by year.

The Wilcoxon test is the most used in the literature, presented in 21% of the collected papers, followed by the Friedman test and Wilcoxon rank-sum test, each studied in 12% of the research. The rest of the statistical tests have been used in less than 10% of the literature. Figure 18 shows the statistical tests studied by year for the tests used in at least three investigations.

### 4.3. What Machine Learning Techniques Have Been Used to Calculate Fitness in the Feature Selection Problem?

In machine learning, classifiers are relevant for data analysis, pattern recognition, and decision-making [188,189,190,191,192]. In order to determine which classifiers are employed in this context, the collected literature was analyzed, allowing us to improve the understanding of the evolution of classifier usage, particularly in evaluating the effectiveness of selected features in various models. The review of these articles uncovers trends, distributions, and characteristics of classifiers as evaluative tools in the feature selection process. Comprehending these aspects is essential to grasp how classifiers enhance the efficiency and accuracy of machine learning models. By examining the prevalence and variations in classifier deployment, we gain insights into how researchers innovatively use these algorithms in complex problems, explicitly selecting and validating features across diverse datasets. The following sections provide a detailed analysis of these trends, elucidating usage patterns and classifier categories.

#### 4.3.1. Classifier Trends over Time

**Figure 19:**
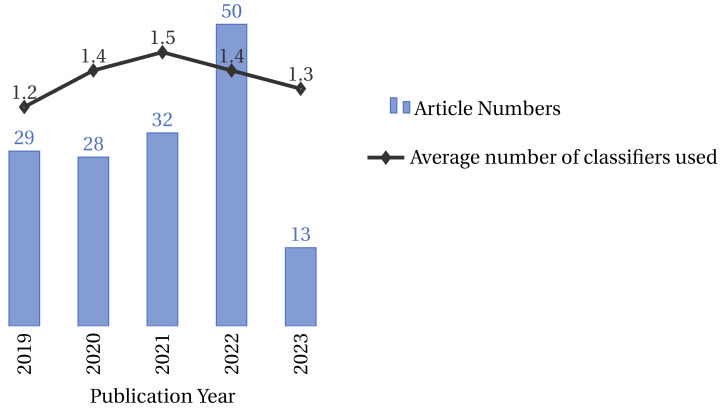
Trend in the average number of classifiers used per article.

Researchers have increasingly deployed classifiers over the past five years in feature selection and optimization tasks. This trend is evident in the rising number of articles published on the topic and the average number of classifiers used per article, as shown in Figure 19. This figure presents two sets of data: the number of articles published per year (represented as a bar chart) and the average number of classifiers used per article (depicted as a line graph). It is important to note that these two data series are represented on different scales to ensure clarity and legibility. While the bar chart reflects the total number of articles analyzed (152 out of 161, excluding 9 articles where the classifier was not specified), the line graph for the average number of classifiers is plotted on a separate scale. This approach was adopted to prevent the average number of classifiers from appearing too close to the zero line, thereby preserving its visibility and explanatory power in the figure. The dual-scale representation, while creating a visual impression of disproportionality, is essential for an accurate and clear depiction of the trends.

This trend is likely due to the increasing popularity of machine learning and the growing availability of data. Classifiers are a powerful tool for extracting insights from data and can be used to improve the performance of feature selection and optimization algorithms. The trend towards using more classifiers per article is also noteworthy. This suggests that researchers are increasingly experimenting with different classifier types and combinations to achieve better results.

Examining these trends, we discern several patterns. Over the years, the increasing number of articles suggests the growing significance of feature selection and optimization in the research landscape. Notably, the slight fluctuations in the average number of classifiers per article indicate the adaptability of the field, where researchers balance the quest for precision with practical considerations.

In this context, there are a particular set of highlight articles that, although not explicitly detailing the classifiers used, made substantial contributions to the field, directing their focus toward innovative methodologies and applications. For instance, Chaudhuri et al. [110] made strides in feature selection using the binary crow search algorithm with time-varying flight length. Long et al. [180] investigated numerical optimization and feature selection through a butterfly-balanced optimization algorithm. In similar veins, the studies in Takieldeen et al. [122] and Kalra et al. [123] introduced the dipper-throated optimization algorithm and a novel binary emperor penguin optimizer, respectively, both serving feature selection tasks. Further contributions came from Tubishat et al. [50], who delved into dynamic generalized normal distribution optimization for feature selection, and Li et al. [187] designed a two-stage hybrid feature selection algorithm with applications in Chinese medicine. Oyelade et al. [144] explored evolutionary binary feature selection using an adaptive Ebola optimization search algorithm tailored for high-dimensional datasets. Meanwhile, a hybrid global optimization algorithm for feature selection was meticulously examined in [186], and a dynamic butterfly optimization algorithm for feature selection was showcased in [151].

While the classifiers in these studies may not be distinctly outlined, the sheer breadth of approaches and applications in these works underscores the diversity and innovation pulsating through feature selection and optimization, offering a rich tapestry of knowledge and avenues for future exploration.

#### 4.3.2. Classifier Usage by Year

**Figure 20:**
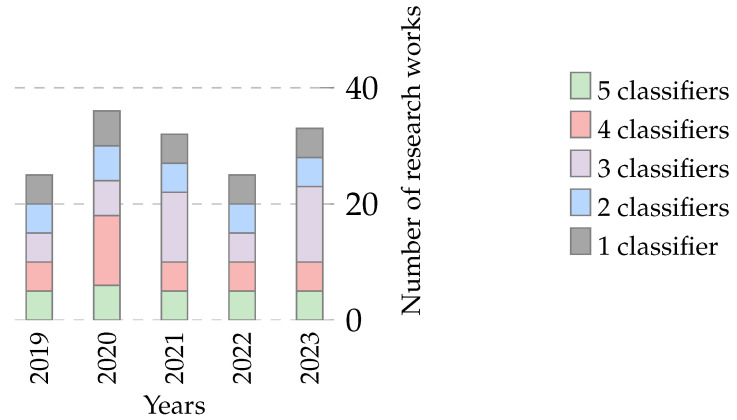
Number of classifiers by year.

Examining the evolution of classifier usage across different years can provide valuable insights into the dynamic landscape of feature selection and optimization. Figure 20 shows the annual distribution of papers based on the number of classifiers employed. This chart provides a comprehensive overview of classifier usage across the years, categorized by the number of classifiers employed in each paper.

In this context we notice the following patterns and shifts in classifier usage over the years:

- **2019**: The majority of articles (86.2%) employed a single classifier [27,28,29,42,44,45,51,54,62,77,78,79,80,81,82,83,84,85,86,87,88,155,163,164,166], setting the tone for a strong emphasis on foundational methodologies.
- **2020**: The trend continues, with the prominence of single classifiers remaining steady at 78.6% [23,31,41,46,53,71,72,89,90,92,93,95,152,153,154,156,157,158,159,165,168,173]. A slight increase in articles employing two classifiers suggests a nascent exploration of combinations [91,167,175].
- **2021**: The year sees an expanded adoption of multiple classifiers, with a noticeable uptick in papers employing three (12.5%) [35,56,107,176] and four (3.1%) classifiers [106]. This potentially signifies a growing confidence in ensemble methodologies.
- **2022**: A significant leap is observed in the total number of articles, accompanied by a proportional increase in the use of diverse classifiers. The rise in articles employing multiple classifiers, including five classifiers [117], underscores a dynamic approach to optimization challenges.
- **2023**: The number of articles decreases, and the distribution reverts to a focus on single classifiers [40,70,143,145,146,148,149,150,160,184,185], while a minimal presence of two and four classifiers persists.

The trends in classifier usage suggest that researchers are moving from foundational exploration to embracing more complex and multifaceted deployments. The increasing adoption of ensemble methodologies aligns with the field’s maturation and the need to comprehensively address complex optimization objectives.

#### 4.3.3. Classifier Descriptions

To make informed decisions about feature selection and optimization, it is essential to understand the nuances of different classifiers. This subsection summarizes the purposes and fundamental characteristics of each classifier. Table 4 presents a concise delineation of each classifier’s intent and attributes.

From the robust support vector machine (SVM) that finds optimal separating hyperplanes, to the intricate multilayer perceptron (MLP) that captures complex nonlinear relationships, each classifier serves a unique role. Ensembles like random forest (RF) and extreme gradient boosting (XGBOOST) showcase the power of collective learning, while naive Bayes (NB) relies on probabilistic reasoning for classification. Decision trees, represented by Decision Tree C4.5 (DT C4.5) and its variants, offer interpretability.

These classifier descriptions provide a quick reference guide for comprehending the diverse methodologies related to feature selection and optimization.

#### 4.3.4. Most Common Classifiers

**Figure 21:**
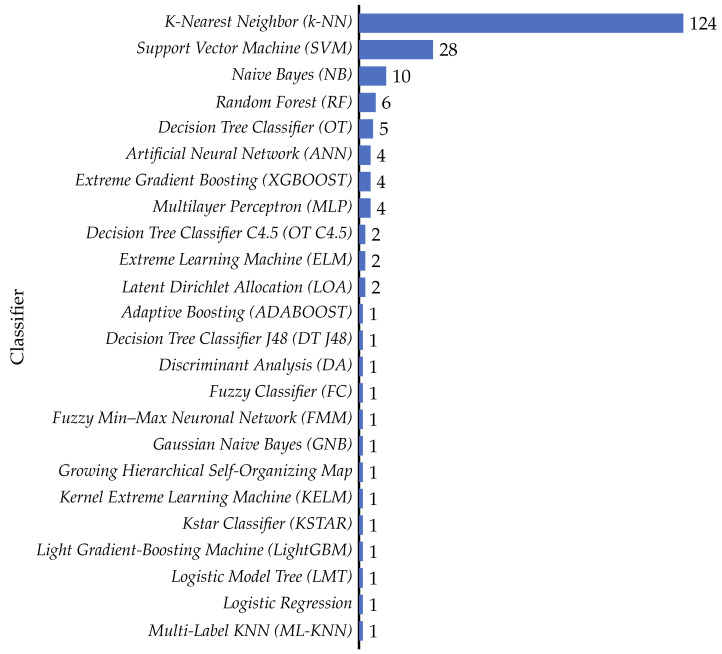
Classifier distribution analysis: count of articles per classifier.

A diverse range of classifiers are used in feature selection and optimization to tackle complex challenges. Figure 21 shows the prevalence of specific classifiers in the reviewed articles.

At the forefront of the classifier ensemble, the k-nearest neighbor (k-NN) emerges as the most frequently employed technique, featured in a substantial 77% of the papers collected [27,28,29,30,32,35,36,37,38,39,42,43,45,46,47,48,49,51,53,54,55,56,57,60,62,64,65,66,67,68,69,70,77,78,79,80,81,82,83,84,85,86,87,88,89,90,91,92,93,95,96,97,99,100,101,102,103,104,105,106,107,108,109,112,113,114,115,116,117,118,119,120,121,124,125,126,127,128,129,130,131,132,133,134,135,136,137,138,139,140,141,142,143,145,146,147,148,149,152,153,154,155,156,157,158,159,160,161,163,165,166,167,168,172,173,174,177,178,179,181]. This exemplifies its role as a foundational and versatile approach in addressing complex optimization tasks. Notably, the support vector machine (SVM) follows, with a notable presence in 17.4% of papers [21,32,35,39,40,43,55,56,57,59,60,72,74,91,106,107,117,147,164,169,171,174,175,176,177,178,181,182]. The presence of naive Bayes (NB) [33,35,60,94,106,172,176,177,178,181] and various decision tree classifiers, including Decision Tree Classifier C4.5 (DT C4.5) [30,94] and random forest (RF) [22,34,147,162,167,176], underscores the ongoing significance of interpretable and ensemble-based methodologies. Meanwhile, emerging techniques like Extreme Gradient Boosting (XGBOOST) [22,33,56,117] and Artificial Neural Network (ANN) [31,71,115,175] reflect the integration of modern learning paradigms to address complex optimization endeavors.

#### 4.3.5. Classifier Categories

**Figure 22:**
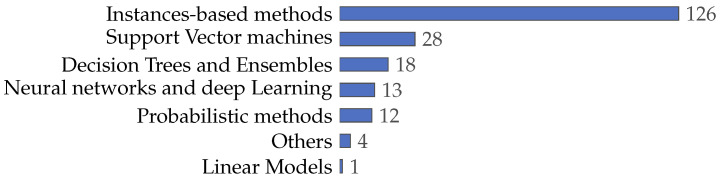
Research by classifier.

This research employed various methodologies, highlighting the range of approaches within the field. Figure 22 enriches our comprehension of classifier diversity and highlights key focus areas.

Dominating the field are instance-based methods [23,27,28,29,30,32,35,36,37,38,39,42,43,45,46,47,48,49,51,53,54,55,56,57,60,62,63,64,65,66,67,68,69,70,77,78,79,80,81,82,83,84,85,86,87,88,89,90,91,92,93,95,96,97,99,100,101,102,103,104,105,106,107,108,109,112,113,114,115,116,117,118,119,120,121,124,125,126,127,128,129,130,131,132,133,134,135,136,137,138,139,140,141,142,143,145,146,147,148,149,152,153,154,155,156,157,158,159,160,161,162,163,165,166,167,168,172,173,174,177,178,179,181,182,183,184] employing algorithms such as k-nearest neighbor (k-NN) and its multi-label variant (ML-kNN). This category stands out for its intuitive logic, classifying new instances by a majority vote among the nearest neighbors, thus encapsulating a local approximation of the target function.

The support vector machines category [21,32,35,39,40,43,55,56,57,59,60,72,74,91,106,107,117,147,164,169,171,174,175,176,177,178,181,182] is characterized by its foundational SVM algorithm. This method seeks the optimal separating hyperplane in a transformed feature space. It is recognized for its prowess in high-dimensional settings, making it a powerful tool for binary and multi-class classification problems.

Decision trees and ensembles [22,30,33,34,56,59,65,94,107,111,117,138,147,162,167,176,181,182] represent a collective of methodologies like the decision tree classifier (including variations such as J48 and C4.5), random forest, and adaptive boosting (AdaBoost). These models are particularly noted for their interpretability and the ensemble strategies that aggregate the predictions of multiple trees to enhance performance and mitigate overfitting.

The neural networks and deep learning approaches [31,44,58,71,75,106,115,117,150,170,174,175,185] encompass models such as artificial neural networks (ANNs), multilayer perceptrons (MLPs), and more advanced configurations like deep learning architectures. These methods simulate the complex interconnections of a biological brain and excel in capturing nonlinear relationships within large datasets.

The probabilistic methods [33,35,41,60,94,98,106,172,176,177,178,181] include naive Bayes (NB), Gaussian naive Bayes (GNB), and multi-label naive Bayes (MLNB). These algorithms are based on applying Bayes’ theorem and are valued for their ability to handle uncertainty and deliver probabilistic predictions.

A category named “other classifiers/algorithms” includes methods not typically aligned with standard classifier frameworks previously described. Discriminant analysis (DA), employed to classify observations into predefined classes based on their features, is discussed in one study [32]. Fuzzy classifiers (FCs), which apply fuzzy logic to handle ambiguous class memberships, are utilized in another work [47]. Additionally, latent Dirichlet allocation (LDA) is used to model latent topics within text corpora, as demonstrated in two articles [73,174].

Lastly, linear models were mentioned in one article [94], with logistic regression being a primary example of this approach. It models the probability of a binary outcome and is often favored for scenarios where relationships between the input variables and the output are presumed to be linear.

### 4.4. What Metaheuristics Have Been Used to Solve the Feature Selection Problem?

Researchers have employed a diverse range of metaheuristics to address the feature selection problem. This section identifies and discusses the various metaheuristics featured in the reviewed articles. Key aspects covered include the frequency of metaheuristic usage, observed binarization approaches, hybridization of metaheuristics, techniques employed to enhance performance, the application of multi-objective approaches in metaheuristics, and the interplay between objective function formulation and metaheuristics.

#### 4.4.1. Frequency of Source Metaheuristics Utilization

**Figure 23:**
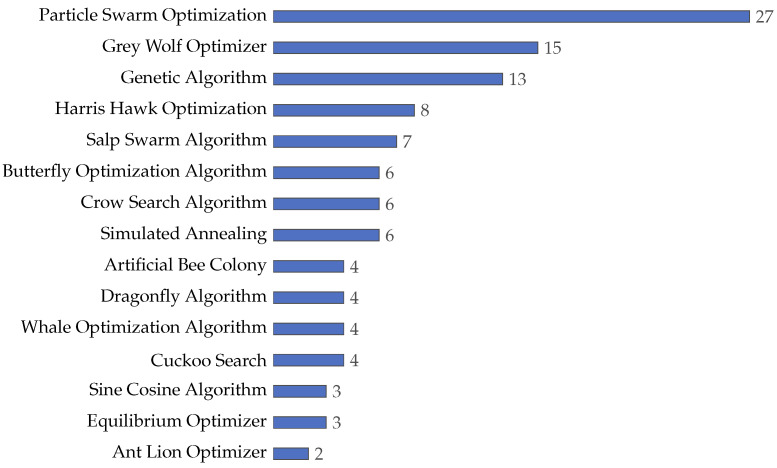
Research by metaheuristic.

Metaheuristics are general-purpose algorithms that, with few modifications, can solve different optimization problems [193]. We refer to “**base**” or “**source**” metaheuristics as the main metaheuristics used by researchers that are later adapted. Figure 23 shows the metaheuristics that have emerged as the most frequently utilized for solving the feature selection problem.

Particle swarm optimization is a population metaheuristic based on the swarming behavior of animals such as birds or fish [194]. This metaheuristic is the most used by the authors in [22,27,28,30,32,35,36,38,39,45,54,65,69,77,79,80,83,87,100,127,138,153,155,161,168,174,186]. The second most used metaheuristic is the grey wolf optimizer, a population metaheuristic based on the hunting behavior of grey wolves [195] and used in [51,71,84,87,88,95,101,105,128,134,137,157,160,170,173]. The other metaheuristic used in more than ten studies is the genetic algorithm, a population metaheuristic inspired by Darwin’s laws of evolution [196] and used in [31,32,41,56,58,63,72,73,79,98,100,135,175].

It is noteworthy that while some metaheuristics like PSO, GWO, and GA have been extensively used, a vast array of other algorithms have been explored less frequently. This diversity suggests that the field of feature selection is rich and continuously evolving, with researchers experimenting with different algorithms to find the most suitable solution for specific problems.

The prominence of these metaheuristics also underscores the importance of continuous improvement and adaptation. The no free lunch theorem [18,19] inspires researchers to innovate. Given this, newer variants or hybrid versions of these algorithms are likely to emerge, further expanding the boundaries of feature selection research.

#### 4.4.2. Binarization Approaches in Metaheuristics

Binarization in metaheuristics refers to converting continuous solutions into binary solutions, which is essential for problems like feature selection where the solution space is binary [20,197]. There are two primary approaches observed in the reviewed documents related to binarization: Binarization in metaheuristics is transforming continuous solutions into binary solutions [20,197], a necessary step for problems like feature selection where the solution space is binary. The reviewed literature indicates two primary binarization approaches:

- **Direct binarization**: This approach involves straightforward methods where the binarization process is direct and does not involve extensive testing or evaluation of different techniques. It is often used for its simplicity and efficiency. Cases of this approach are the papers [21,35,36,40,48,63,67,73,80,82,87,94,95,98,101,104,105,108,112,115,118,119,123,133,134,136,149,150,152,153,158,159,161,169,177].
- **Binarization with various approaches**: This approach involves a comprehensive study and evaluation of multiple binarization techniques to determine the most effective one for a given problem. It is more exhaustive and aims to find the optimal binarization method for specific scenarios. Cases of this approach are the articles [47,71,78,89,91,92,96,106,107,110,114,124,129,131,132,138,142,144,146,154,165,172,176,179].

**Figure 24:**
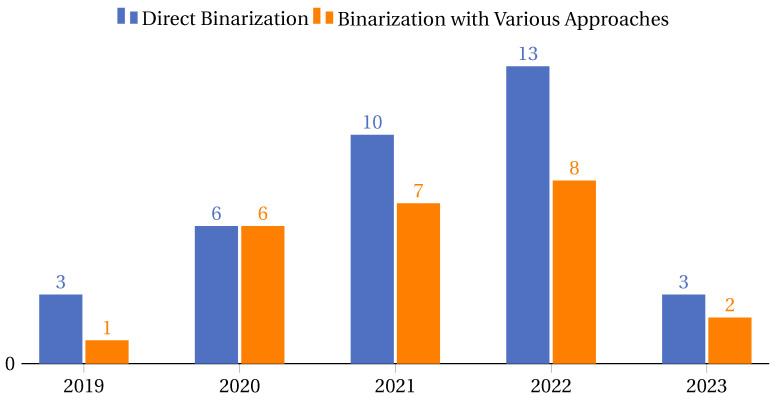
Direct Binarization vs. Binarization with various approaches by year.

Figure 24 shows the comparative trends over the past five years for these binarization approaches.

Analyzing Figure 24, it is evident that direct binarization has significantly increased from 2019 to 2022. This could be attributed to its straightforward nature, making it a preferred choice for researchers who prioritize efficiency. On the other hand, binarization with various approaches has remained relatively consistent over the years, with a slight increase from 2019 to 2022. This indicates a steady interest in exploring and evaluating different binarization techniques to find the most effective one.

#### 4.4.3. Hybridization in Metaheuristics

**Figure 25:**
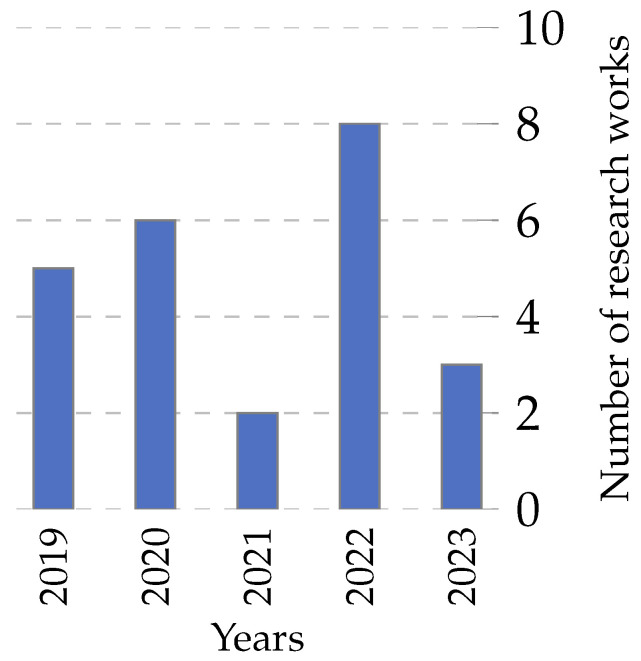
Metaheuristic hybridization.

Hybridization in the context of metaheuristics refers to combining two or more metaheuristic algorithms to create a new, often more efficient, method. This subsection specifically focuses on hybridization, which involves merging one metaheuristic with another, excluding combinations with non-metaheuristic techniques. The primary objective behind such hybridization is to capitalize on the strengths of the individual metaheuristics while mitigating their weaknesses. By integrating the best features of multiple metaheuristics, these hybrid methods often achieve superior performance, faster convergence, and more robust solutions, especially in complex optimization problems like feature selection. Figure 25 shows the trend in metaheuristic hybridization over the past five years.

The data show a noticeable fluctuation in the interest in metaheuristic hybridization over the years. While there was a steady increase from 2019 [85,86,87,88] to 2020 [72,92,154,157,159,173], there was a significant drop in 2021 [97,161]. However, 2022 [69,112,113,121,135,136,137,140] saw a resurgence in the number of articles focusing on hybridization, indicating a renewed interest or perhaps the emergence of new hybrid techniques that garnered attention. The decline in 2023 [70,144,145], similar to other trends, might be attributed to the data only covering up to April, and it is possible that the numbers might increase as the year progresses.

The fluctuating trend suggests that while hybridization remains a topic of interest, its application and exploration might be influenced by various factors, including the emergence of new standalone algorithms, the complexity of hybrid methods, or shifts in research focus. Nonetheless, the consistent presence of hybridization articles underscores its importance and potential in enhancing feature selection methodologies.

**Figure 26:**
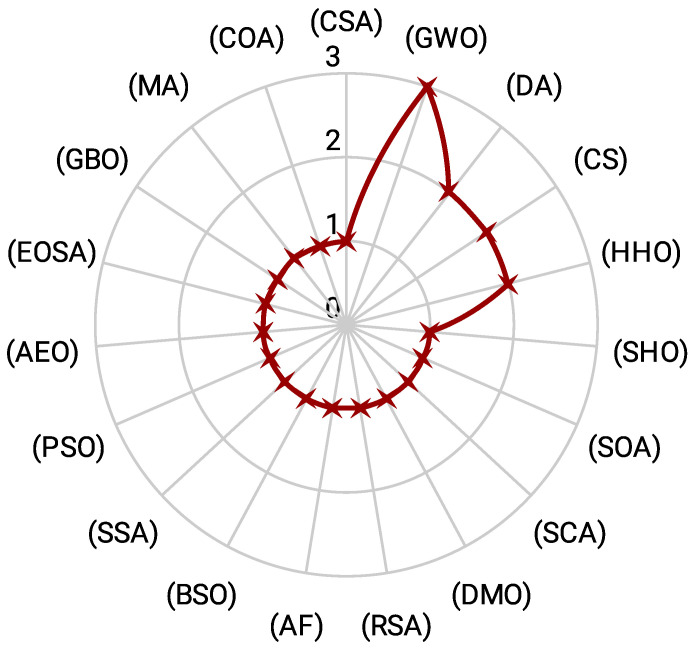
Frequency of foundational metaheuristics.

In the hybridization process involving two metaheuristics, one typically serves as the foundational or base algorithm, while the other acts as an enhancement. This enhancement specifically targets and strengthens aspects of the base metaheuristic that may be perceived as weaker than other metaheuristics. From 2019 to April 2023, a review of 24 articles revealed various metaheuristics employed as foundational or base algorithms in the hybridization process. These metaheuristics serve as the backbone upon which enhancements are made using other algorithms to address specific weaknesses or to leverage unique strengths. Figure 26 shows the various metaheuristics employed as foundational algorithms in the hybridization process from 2019 to April 2023.

Analyzing Figure 26 we detect the following:

- The grey wolf optimizer (GWO) [87,88,157] stands out as the most frequently used foundational metaheuristic, having been employed as a base in three different studies. This suggests its prominence and potential adaptability in hybrid models.
- The dragonfly algorithm (DA) [97,155], cuckoo search (CS) [113,173], and harris hawk optimization (HHO) [140,159] have each been utilized twice. Their repeated use indicates their significance and robustness as foundational techniques in the hybridization process.
- Most of the metaheuristics, including but not limited to the spotted hyena optimization algorithm (SHO) [85], seagull optimization algorithm (SOA) [86], sine cosine algorithm (SCA) [92], and dwarf mongoose optimization (DMO) [112], have been used once as foundational algorithms. This showcases the diversity of metaheuristics explored by researchers in the hybridization process.
- The wide range of foundational metaheuristics, even those used just once, underscores the richness of the field. It indicates that researchers continuously experiment with different base algorithms to find the most suitable combinations for specific problems.

**Figure 27:**
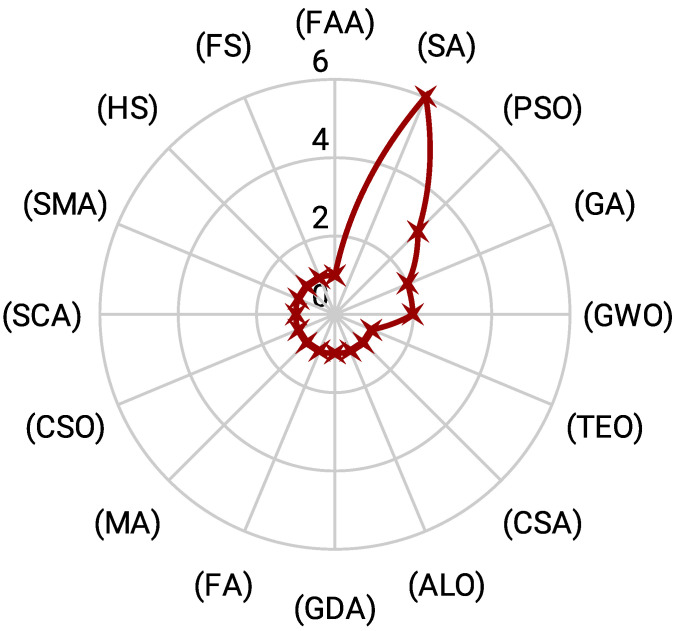
Frequency of metaheuristics enhancers.

Figure 27 visually represents the various metaheuristics employed to enhance or improve base algorithms. These enhancers are specifically used to address specific weaknesses in the base metaheuristics or to capitalize on unique strengths.

Analyzing Figure 27, the following insights can be derived:

- Simulated annealing (SA) [85,97,112,121,144,159] emerges as the most frequently used metaheuristic for enhancement, with a count of six. Its recurrent use suggests it offers versatile capabilities in refining and optimizing base metaheuristics.
- Particle swarm optimization (PSO) [87,155,161] has been employed three times as an enhancer, indicating its adaptability and effectiveness in improving various foundational algorithms.
- The genetic algorithm (GA) [72,135] and Grey Wolf Optimizer (GWO) [137,173] have both been utilized twice as enhancing metaheuristics. Their repeated use underscores their potential in augmenting the performance of base algorithms.
- A wide array of metaheuristics, including the firefly algorithm (FAA) [144], thermal exchange optimization (TEO) [86], the cuckoo search algorithm (CSA) [88], and harmony search (HS) [154], among others, have been used once. This diversity reflects the rich experimentation in the field, with researchers exploring various combinations to achieve optimal results.

#### 4.4.4. Techniques to Enhance Metaheuristics

**Figure 28:**
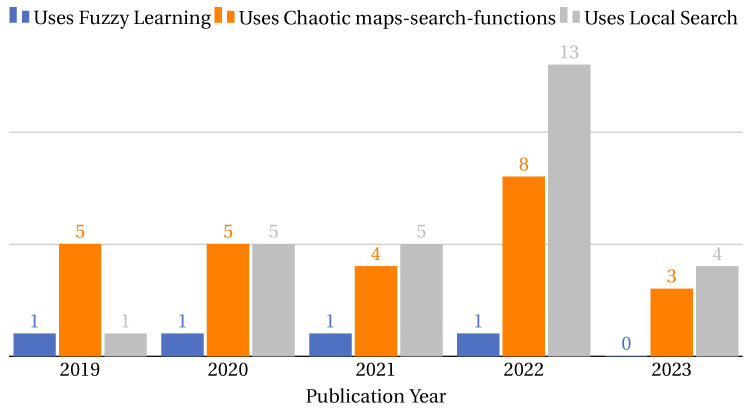
Enhancement techniques trend.

Researchers have developed various techniques to enhance the performance of metaheuristics for feature selection. The most commonly used techniques include chaotic maps, local search, and fuzzy learning. These techniques are used to improve the exploration and exploitation capabilities of metaheuristics, which can lead to better solutions. It is important to note that these are just a few of the many techniques researchers have used to optimize metaheuristics. As the field of feature selection continues to evolve, researchers are likely to develop even more innovative and effective techniques. Figure 28 illustrates the trend of techniques utilized over the analyzed time period.

From Figure 28, several observations can be made:

- Chaotic maps search function: With a total of 25 instances across the years [34,40,42,53,81,90,93,103,106,114,117,120,121,126,129,134,143,147,156,159,161,162,163,164,183] this technique has seen consistent use, with a noticeable peak in 2022. Its application suggests that researchers find value in its chaotic dynamics to enhance the exploration capabilities of metaheuristics.
- Local search: This technique has been the most frequently employed, with a total of 28 instances [36,40,46,50,69,77,93,99,103,107,111,112,113,120,126,127,128,129,130,134,143,145,147,151,153,161,174,183]. Particularly in 2022, there was a significant surge in its application, indicating its effectiveness in refining solutions and improving convergence rates.
- Fuzzy learning: While this has been used less frequently, with only four instances over the years [36,44,47,53], it offers a unique approach to handling uncertainties and improving adaptability in metaheuristics.

In conclusion, while chaotic maps, local search, and fuzzy learning are among the more common techniques to enhance metaheuristics, their varied application over the years underscores the dynamic nature of research in this field. Researchers continuously experiment with different techniques, seeking the most effective combinations to address complex optimization challenges.

#### 4.4.5. Multi-Objective Approaches in Metaheuristics

Multi-objective metaheuristics are specifically designed to tackle problems with multiple objectives. This is important because many real-world problems have multiple conflicting objectives, and single-objective metaheuristics cannot find optimal solutions for these problems [198].

**Figure 29:**
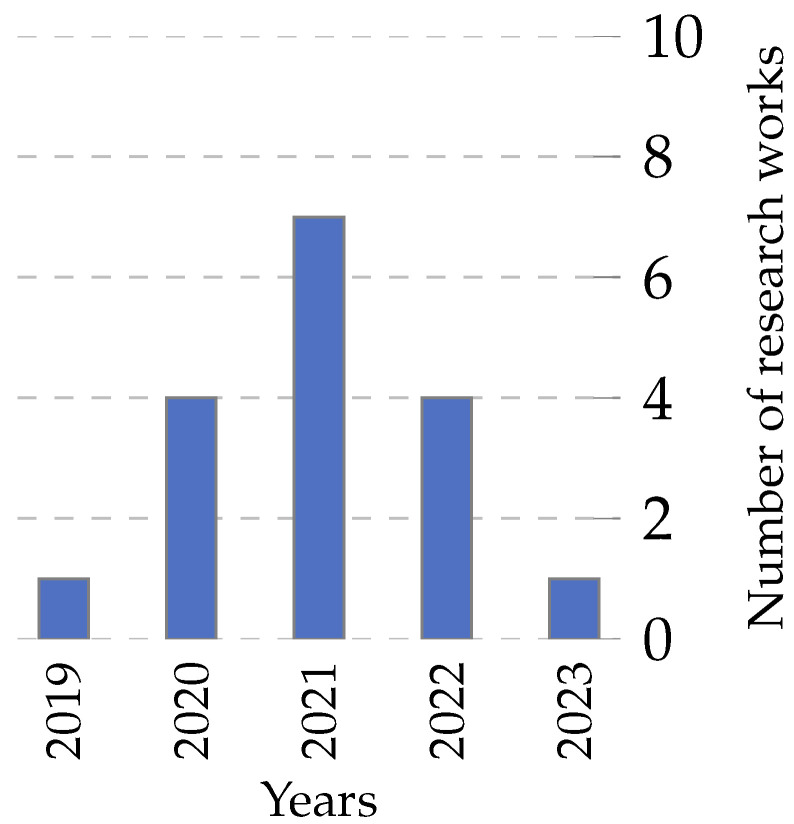
Multi-objective metaheuristics.

Multi-objective metaheuristics aim to find solutions that balance and optimize all of the objectives simultaneously [198]. This is a challenging task, but it is essential in many real-world applications. Figure 29 shows the evolution of multi-objective metaheuristics proposals over the past five years. This trend suggests a growing interest in multi-objective metaheuristics, likely due to the increasing complexity of real-world problems.

From the data, several observations can be made:

- There was a noticeable increase in multi-objective metaheuristic proposals from 2019 to 2021, peaking in 2021 with seven proposals [63,64,65,66,73,74,179]. This suggests a growing recognition of the importance of multi-objective approaches during this period.
- The numbers in 2022 and 2023 (up to April) show a decline, which could be attributed to various factors, including shifts in research focus or the maturation of multi-objective techniques developed in previous years.
- In the context of feature selection, multi-objective metaheuristics are invaluable. Feature selection often involves balancing reducing dimensionality (and thus computational cost) and retaining the most informative features for accurate prediction or classification. Multi-objective approaches provide a framework to navigate these conflicting objectives, ensuring robust and efficient models.

Table 5 presents an overview of the above-mentioned algorithms, where we highlight their primary areas of application, fundamental innovation, and the results of their respective evaluation processes.

#### 4.4.6. Relationship between Objective Function Formulation and Metaheuristics

**Figure 30:**
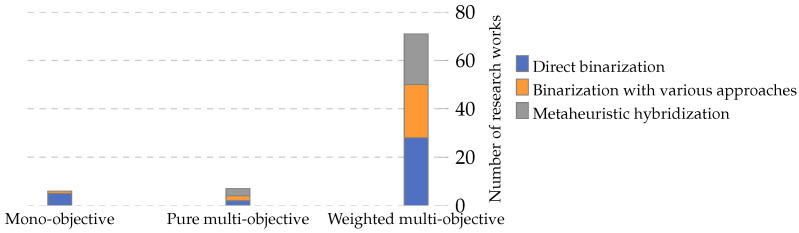
Objective function formulation and metaheuristic (hybridization and binarization).

The relationship between the formulation of the objective function and the chosen metaheuristic offers a lens into the evolving research preferences and trends in feature selection. Figure 30 elucidates this relationship, detailing the distribution of articles based on the objective function and the metaheuristic employed.

The majority of articles with a “weighted multi-objective” formulation predominantly employ both “direct binarization” [73,80,82,87,94,95,98,101,104,105,108,112,115,118,119,123,133,134,136,149,150,152,153,158,159,161,169,177] and “binarization with various approaches” [78,89,91,92,96,106,107,110,114,124,129,131,132,138,142,144,146,154,165,172,176,179], with a similar inclination towards “metaheuristic hybridization” [85,86,87,88,92,97,112,113,121,135,136,137,140,144,145,154,155,157,159,161,173].

Interestingly, “pure multi-objective” formulations, while having a limited presence in “direct binarization” [63,67] and “binarization with various approaches” [71,179], exhibit just a slight edge towards “metaheuristic hybridization” [69,70,72].

Conversely, “mono-objective” formulations show a strong favor for “direct binarization” [21,35,36,40,48], with minimal exploration of other metaheuristics. This distribution underscores a discernible trend: researchers increasingly lean towards weighted multi-objective formulations when delving into diverse metaheuristics, likely due to the adaptability and robustness these formulations provide in tackling intricate feature selection challenges.

### 4.5. Which Datasets Are Commonly Used as Benchmarks, and Which Are Derived from Real-World Applications?

This section offers a detailed analysis of dataset usage across the reviewed articles. It includes an overview of the commonality of benchmark datasets, their real-world applications, and instances where they are combined. The selection of datasets and their sources is pivotal in ensuring research efficacy and relevance, especially in feature selection and metaheuristics. We thoroughly examine the most frequently used datasets, elaborating on their origins, characteristics, and distinct features. Further, this section shows the repositories from which these datasets are sourced, equipping readers with a comprehensive understanding of the data landscape integral to the studies under review.

#### 4.5.1. Overall Trend in Dataset Usage

**Figure 31:**
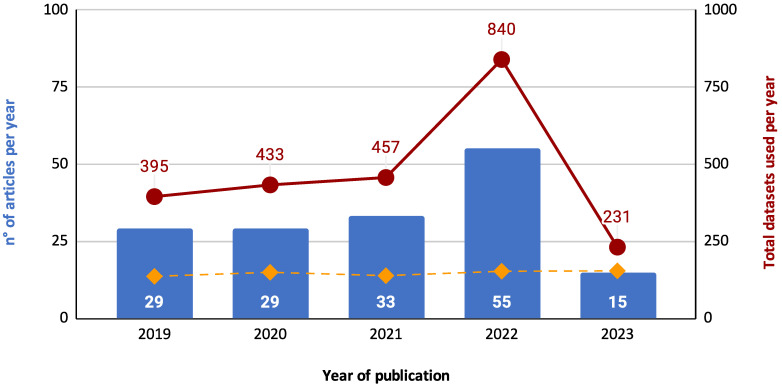
Dataset utilization and article publications trends over time.

Figure 31 shows the trend in dataset usage across articles focused on feature selection and metaheuristics over five years, from 2019 to 2023. The chart is segmented by the year of publication, indicating the number of articles produced each year, the total datasets utilized, and the average number of datasets employed per article. From the data, in 2019, 29 articles made use of a combined total of 395 datasets, averaging 13.62 datasets per article. The following year, 2020, also witnessed 29 articles. However, there was an increase in dataset usage, with a cumulative count of 433, translating to an average of 14.93 datasets for each article. 2021 experienced a slight increase in the number of articles to 33, utilizing 457 datasets in total. The mean datasets per article stood at 13.85. A significant surge was noted in 2022, with 55 articles being published. These articles utilized 840 datasets, averaging 15.27 datasets per article. As of the current year, 2023, data from 15 articles has been analyzed. These articles have used 231 datasets, leading to an average of 15.40 datasets per article, slightly higher than the previous year.

**Figure 32:**
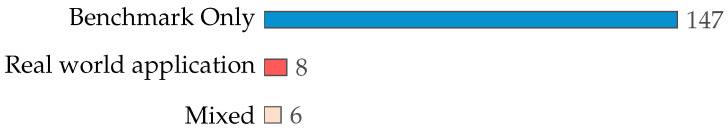
Distribution of benchmark vs. real-world applications in the articles.

Building upon the dataset usage trends, Figure 32 offers a deeper exploration into the nature of these datasets, categorizing them as ‘benchmark only’, ‘real-world application’, or a combination of ‘both benchmark and real-world applications’. The authors consider “benchmark” those datasets that were described as such in the articles reviewed or that are typically described as such in the literature. On the other hand, “real-world application” datasets were constructed specifically for the article in question or from dataset repositories whose purpose is to provide datasets for researchers to conduct investigations on these datasets.

From the data presented in Figure 32, a predominant trend emerges related to the utilization of benchmark datasets [27,28,29,30,31,32,33,34,36,37,38,39,40,41,42,43,46,47,48,49,50,53,54,55,56,57,58,59,60,62,63,64,65,66,67,68,69,70,71,72,73,74,75,77,78,79,80,81,82,84,85,86,87,88,89,90,91,92,93,94,95,96,97,98,99,100,101,102,103,104,106,107,108,109,110,111,112,113,114,115,116,117,118,119,120,121,122,123,124,125,126,127,128,129,130,131,132,133,134,135,136,137,138,139,140,142,143,144,145,146,148,149,150,151,152,153,154,155,156,157,158,159,160,161,162,163,165,166,167,168,169,170,171,172,173,174,175,176,177,179,180,181,182,183,184,185,186]. This underscores the preference for controlled, standardized datasets that allow comparative analysis across different feature selections and metaheuristics. In contrast, a smaller subset of eight articles focused solely on real-world application datasets [21,35,45,51,83,105,164,178]. These articles prioritized practical, real-world implications and the challenges that come with them. A nuanced approach was seen in six articles, which employed both benchmark and real-world datasets [22,23,44,141,147,187]. This suggests a comprehensive methodology that balances the theoretical robustness of benchmark datasets with the practical relevance of real-world data.

This distribution highlights the prevailing inclination towards benchmark datasets in the domain. However, the existence of papers using real-world datasets or combining both suggests a budding recognition of the importance of grounding research in real-world scenarios and challenges.

#### 4.5.2. Real-World Application Datasets and Their Characteristics

Following the analysis presented in Figure 32, this subsection delves into the specifics of datasets employed in real-world application studies. These studies, though fewer in number compared to those using benchmark datasets, provide crucial insights into the application of machine learning in practical settings.

- The authors in [178] utilized a dataset constructed from the Twitter API focusing on cancer and drugs, enabling sentiment analysis and text classification.
- For industrial maintenance, The authors in [44] employed a dataset designed for motor fault detection.
- The authors in [164] involved a dataset of 553 drugs bio-transformed in the liver, annotated with toxic effects such as irritant, mutagenic, reproductive, and tumorigenic, each represented by chemical descriptors.
- The authors in [45,51,83] made significant use of the NinaPro database. This database provided EMG signals from healthy subjects and amputees, covering various hand and wrist motions essential for prosthetics and rehabilitation research.
- The authors in [23] used hyperspectral image datasets and spectral data of typical surface features, indicating the application of machine learning in environmental monitoring.
- The authors in [35], a dataset of 500 Arabic email messages from computer science students was analyzed, showing machine learning’s application in language processing and cybersecurity.
- The authors in [21] examined data from Iraqi cancer patients, offering a comprehensive dataset for healthcare research across multiple cancer types.
- The authors in [22] focused on constructing a dataset from Zeek network-based intrusion detection logs, underscoring machine learning’s role in network security.
- The authors in [187] presented a dataset related to medical treatment for cardiogenic shock, highlighting the intersection of machine learning and medical research.
- The authors in [141,147] demonstrated the versatility of machine learning in biological and medical research using datasets from NCBI and brain imaging datasets for disease classification.

These datasets represent a shift towards employing machine learning in diverse and practical scenarios, extending beyond the controlled conditions of benchmark datasets. The use of real-world data challenges the algorithms’ robustness in less predictable environments and ensures the relevance and applicability of machine learning solutions in addressing real-world problems. In reviewing the documentation of these datasets, it becomes evident that clarity in their descriptions is paramount, especially for replicability and application of the research. Benchmark datasets often benefit from well-established documentation practices, clearly outlining their structure in terms of instances, features, and labels. Conversely, some real-world application datasets may have less clear descriptions, particularly those that are custom-constructed or adapted. This is especially challenging in datasets related to text classification, where the complexity of textual data can lead to an extensive range of features. While these documentation discrepancies do not diminish the value of the research, they highlight an opportunity for enhancing reporting standards. Improved clarity and completeness in dataset descriptions would greatly benefit the field by fostering transparency, facilitating study replication, and enhancing the applicability of research findings in real-world scenarios.

#### 4.5.3. Prevalent Datasets and Their Defining Characteristics

Specific datasets have emerged as particularly influential, often serving as foundational benchmarks for multiple studies. By their comprehensive nature or unique characteristics, these datasets have become cornerstones for researchers, enabling rigorous testing and validation of methodologies. Next, we focus on spotlighting the datasets that have been instrumental in feature selection and optimization research. We will outline the most frequently used ones, detailing their source name, subject area, number of instances/samples, features/characteristics, classes/labels, and the repositories or platforms where they can be accessed.

**Figure 33:**
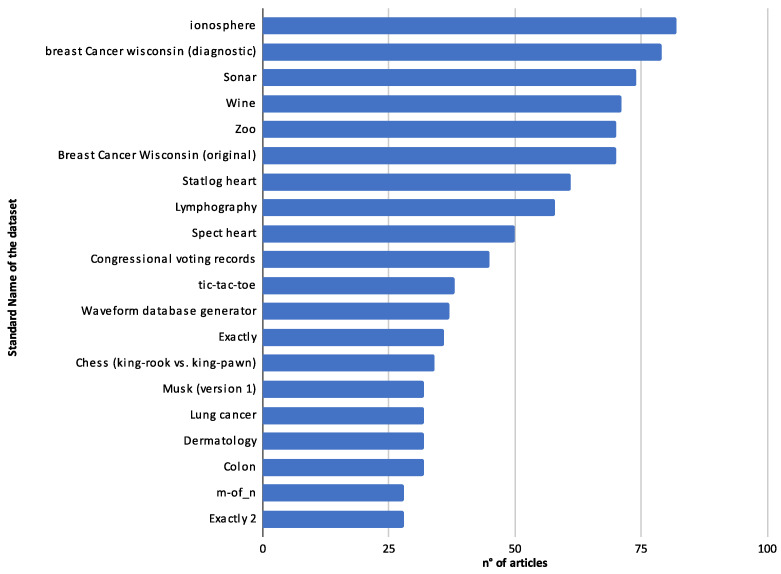
Frequency of dataset usage as a benchmark: top 20.

Figure 33 enumerates the twenty most referenced datasets in the articles under review. The “ionosphere” dataset emerges at the top with 82 mentions [27,29,37,39,40,42,43,44,46,47,50,54,55,60,62,64,65,66,68,70,71,77,78,79,80,82,84,85,86,87,88,89,90,91,92,94,95,97,100,102,104,106,107,108,109,111,112,113,115,118,119,120,124,125,127,130,132,134,135,136,138,139,142,143,145,146,151,152,153,154,155,157,158,160,161,166,167,169,179,180,181,185], closely trailed by the “Breast Cancer Wisconsin (Diagnostic)” dataset at 76 citations [27,28,29,37,43,47,53,55,60,64,65,71,73,77,79,81,82,84,86,87,88,89,91,94,95,96,97,99,101,103,106,107,108,109,111,112,113,114,115,118,119,121,123,127,128,129,130,132,133,134,135,136,138,139,142,143,144,145,147,149,152,153,154,155,158,159,160,161,163,169,173,179,180,181,183,186]. Datasets like “Sonar” [23,27,29,34,37,39,40,42,43,44,46,47,50,54,60,62,65,68,70,71,77,79,81,82,84,86,87,88,89,91,92,94,95,97,98,100,102,104,107,108,109,110,111,112,113,114,115,118,119,127,130,132,135,136,138,139,142,143,144,145,147,150,151,152,153,154,155,158,160,162,166,180,181,185], “Wine” [23,29,37,39,42,47,54,57,64,65,66,68,71,77,78,79,82,84,86,87,89,90,91,92,93,94,95,97,102,103,104,106,107,108,109,110,111,112,118,119,120,123,124,125,127,129,130,132,134,135,136,137,138,139,142,143,144,145,148,149,152,154,155,158,160,161,166,179,180,183,185], and Zoo [23,37,43,59,62,65,66,71,75,78,79,81,82,84,85,87,88,89,90,91,92,94,95,97,100,102,103,104,106,107,108,109,110,111,112,115,118,119,120,123,124,125,129,130,132,134,135,136,137,138,139,142,143,144,145,148,150,152,153,154,155,157,158,160,163,166,167,180,183,185] also secured noteworthy positions, with each being cited at least in 70 articles. The figure shows the most influential and recurrent datasets in feature selection and optimization research, offering insights into the datasets’ prevalence and importance in the academic discourse.

Looking deeper, out of the top ten datasets enumerated in Figure 33, seven pertain to the medical or biological domains. This dominance underscores the significant role of medical and biological data in feature selection and optimization research, possibly due to healthcare data’s complexity, relevance, and critical nature. Datasets such as “Ionosphere” and “Sonar” suggest a diverse application of feature selection techniques across varied fields. However, the prevalence of health-related datasets in the top ranks highlights the growing importance and challenges associated with medical data analytics.

Table 6 provides a detailed breakdown of the 20 most commonly utilized datasets. Each dataset is systematically categorized by:

- Source name: The standardized name or label of the dataset.
- Subject area: The domain or field from which the dataset originates, which reveals a significant leaning towards the medical and biological areas but also showcases diversity, with datasets from physical science, politics, games, and synthetic sources.
- Instances/samples: The number of individual data points or samples in each dataset.
- Features/characteristics: The number of attributes or characteristics each sample in the dataset has.
- Classes: The number of unique labels or outcomes into which the samples can be categorized.
- Reference: Based on DOI, a digital object identifier that provides a persistent link to a dataset.
- Repository: The platform or database from which the dataset can be accessed.

From a glance, it is evident that while datasets like ‘Ionosphere’ and ‘Sonar’ have fewer instances but a moderate number of features, datasets like ‘Colon’ stand out, with many features. The predominance of the UCI repository highlights its role as a primary hub for academic datasets. Moreover, the diversity in subject areas—from ‘physical science’ to ‘game’—underscores the wide applicability and versatility of feature selection and metaheuristics across various fields. It is important to note that within Table 6 three datasets originate from the UCI or ASU repositories, yet they could not be located within those repositories or others. This discrepancy is indicated by ‘n.a.’ (not available) or ‘n.d.’ (not determined) appearing in some table cells, suggesting that access to the information from the source repository is either restricted or unavailable.

#### 4.5.4. A Glimpse into Data Sources

**Figure 34:**
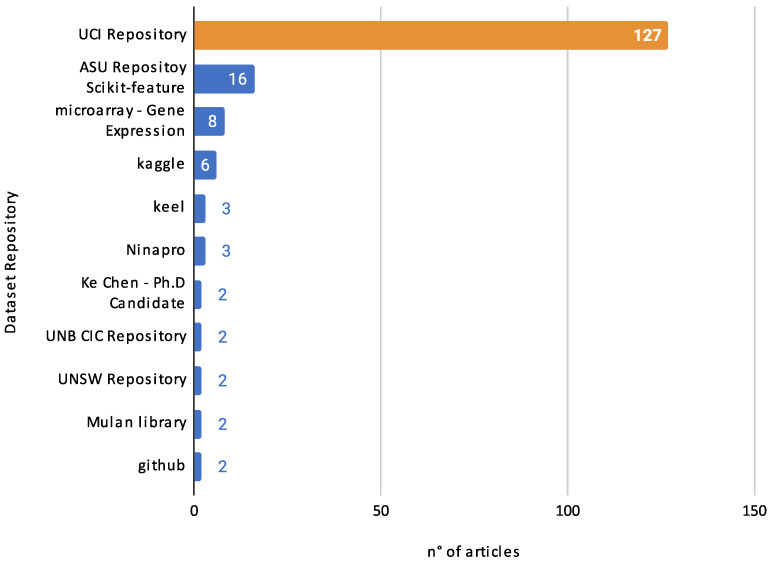
Distribution of articles across popular dataset repositories.

Specific repositories consistently emerged as favored choices for researchers in feature selection and optimization. Figure 34 presents a distribution of these articles across the most popular dataset repositories.

1. UCI Repository: Standing as a stalwart in the academic community, the UCI Repository was referenced by a substantial 127 articles [21,23,27,28,29,31,34,36,37,38,40,41,42,44,45,46,48,49,51,53,54,58,62,63,64,66,67,68,69,70,71,72,73,74,75,77,78,79,80,81,82,83,84,85,86,87,88,89,90,92,93,95,96,97,98,99,100,101,102,103,104,105,108,109,111,112,113,114,116,118,119,120,121,124,125,126,127,128,129,130,131,132,133,134,135,136,137,139,140,141,142,143,145,146,148,149,150,152,153,154,155,156,157,158,159,160,161,163,164,165,166,168,169,170,171,173,179,183,184,185]. A testament to its vast collection and diverse range of datasets, UCI has proven to be an indispensable resource.
2. ASU and Scikit-feature Repositories: Grouped together, these repositories were mentioned in 16 articles [30,36,38,48,49,55,60,67,104,112,116,125,126,156,165,184]. Recognized for specific types of datasets, these platforms offer specialized data that cater to specific niche research areas.
3. Microarray-Gene Expression Datasets: This repository, with a focus on gene expression data, was mentioned in eight articles [65,77,96,116,117,124,154]. It underscores the increasing interest in genomic data and their importance in feature selection studies.
4. Kaggle: A platform widely known for its machine learning competitions, Kaggle also houses an extensive array of datasets. It was cited in six of the reviewed articles [40,131,133,147,148,187].
5. KEEL: With three mentions [40,47,184], the KEEL repository, which emphasizes evolutionary algorithms and data mining, has a defined user base in our set of articles.
6. Ninapro: Also receiving three mentions [45,51,83], Ninapro, which specializes in hand and finger movements, signals its relevance in biomechanical studies.
7. Miscellaneous repositories: Several repositories mentioned in two articles were Ke Chen—Ph.D. Candidate Datasets Repository [36,38], which caters to specific academic projects; the UNB CIC [34,56] and UNSW Repositories [22,56], known for cybersecurity and network datasets; and the Mulan Library [41,63], emphasizing multi-label learning datasets.

While these top ten repositories encompassed the majority of citations, a range of other repositories were also sourced, albeit less frequently. Although not as dominant, these repositories contribute uniquely to the mosaic of datasets available to researchers. The diversity of repositories indicates the breadth and depth of research in feature selection and optimization, with datasets ranging from political records to intricate genomic data.

To facilitate easy access to the various data repositories referenced, Table 7 compiles a comprehensive list of these resources, complete with functional web links verified at the time of writing this article. The aim is to provide a handy, accessible directory of these repositories, ensuring researchers can efficiently retrieve datasets. In the table, the commonly utilized names for the sources of the datasets are systematically presented based on the information reported in the articles. A webpage is provided alongside each name, derived from the citations or references noted in the articles. The third column of the table details the primary use of each repository. The authors make a clear distinction: ’benchmarks’ implies that the repository is predominantly used as a source of datasets frequently employed in machine learning benchmarks and related domains. These datasets serve as standard tests or evaluations for algorithms and models, facilitating comparisons across methodologies.

On the other hand, ‘real-world applications’ signify that the repository is rich in datasets primarily derived from authentic, real-world problems, spanning fields such as medicine, biology, cybersecurity, and more. These datasets are provided to the academic and research communities to foster research that can lead to tangible improvements in human life. They present unique challenges and opportunities for innovation, aiming to contribute to developing solutions or new methodologies that can significantly enhance the quality of life and address pressing real-world issues. All links shown in the Source column of Table 7 were last accessed on 20 December 2023.

### 4.6. Closing of Discussions

This section synthesizes the findings from the literature review, addressing the five research questions that guided our investigation of the feature selection problem.

- Objective function formulation (RQ1): Our review revealed a diversity of objective functions used in feature selection, generally classified as single-objective or multi-objective functions. We observed that while single-objective functions focus on optimizing a single criterion, multi-objective functions, including pure and weighted types, cater to multiple criteria simultaneously. Weighted multi-objective functions were more prevalent in our dataset, suggesting their broader applicability in complex scenarios.
- Performance metrics (RQ2): We classified the performance metrics used in feature selection research into four main categories: classifier metrics, metaheuristic metrics, feature metrics, and statistical tests. Classifier metrics are the most frequently used, emphasizing the importance of the machine learning technique’s performance. The significant use of metaheuristic metrics and feature metrics underscores the complexity of evaluating feature selection methods.
- Used machine learning techniques (RQ3): We investigated machine learning techniques that are improved by feature selection. We found that a variety of classifiers are used, with k-nearest neighbor (k-NN) being the most common. The prevalence of techniques such as SVM, naive Bayes, and decision tree classifiers, including DT C4.5 and random forest, illustrates the wide applicability of feature selection across different learning paradigms.
- Metaheuristics (RQ4): Our study highlights the significant role of metaheuristics in feature selection, particularly particle swarm optimization (PSO), grey wolf optimizer (GWO), and genetic algorithm (GA). Their frequent use points to a preference for adaptive, population-based algorithms adept at handling the complex aspects of feature selection. This observation not only confirms the effectiveness of these methods but also suggests promising directions for future research in enhancing feature selection procedures.
- Practical applications and trends (RQ5): Our analysis of dataset usage trends in feature selection research reveals a slight increase in the number of datasets used per article over time. This shift, along with the dominant use of benchmark datasets and a focus on real-world applications, reflects the escalating complexity and practical significance of feature selection studies. The variety of dataset sources, especially the frequent citation of the UCI Repository, demonstrates the extensive applicability of feature selection in diverse domains.

## 5. Conclusions

In this work, we were able to evidence of the complex and broad field of research related to feature selection. Metaheuristics are algorithms that play a significant role in different Combinatorial Optimization Problems such as Set Covering [216,217,218,219,220], Knapsack Problem [221,222] and Cell Formation Problem [223]. There is also a high interest in hybridizations and modifying native metaheuristics. This is due to the No Free Lunch Theorem, which allows for continued research into new ways of improving metaheuristics.

In this work, we have found that the problem of feature selection has been constantly changing and challenging to be solved with metaheuristics. Given this, we propose a robust evaluation process tailored to compare the effectiveness of various combinations of methods. This process is based on a standardized framework that encompasses three fundamental components:

- Selection of Objective Function: It is interesting to note that the same optimization problem can be represented through three different types of objective functions, each increasing the complexity of the problem. For researchers who are just starting in the field of feature selection, we recommend starting by solving the problem from a single-objective perspective, then moving on to weighted multi-objective, and finally to pure multi-objective.
- Selection of Evaluation Metrics: Regarding metrics, we can observe that there are 4 major groups which are classifier metrics, metaheuristic metrics, feature metrics, and statistical tests. For robustness in future research, we recommend incorporating at least one metric from each of the reported categories.
  − For classifier metrics, we recommend using Accuracy, Error Rate, Precision, Recall, and F-score.
  − For the case of metaheuristic metrics, we recommend using the computational time, the fitness in the case of using a mono-objective function or weighted multi-objective function, and the hyper-volume metric in the case of using a pure multi-objective function.
  − In the case of feature metrics, we recommend reporting the number of features selected and which features were selected.
  − For the case of statistical test, we recommend advocating for a balanced application of both non-parametric tests, such as the Wilcoxon and Friedman tests, and parametric tests like the T-test, supplemented by rigorous post hoc analyses for in-depth insights
  A metric that, in our opinion, should be included in all research is indicating the solution vector, that is, indicating which features were selected by the metaheuristics.
- Selection of classifier: The choice of classifier will depend closely on the dataset used where the important issues to be considered are the unbalance of the target classes, whether it is multi-class or binary-class, and the number of samples. In this sense, we recommend experimenting with more than one classifier to express robust results and can use the KNN, Random Forest, or Xgboost.
- Selection of Benchmark Dataset: Guided by a curated list of the top 20 datasets, ensuring that experimentation and comparison are grounded in both established and innovative contexts.

This proposed framework aims to standardize and elevate the comparative analysis in feature selection and metaheuristics research, fostering a more consistent, transparent, and replicable approach in future studies.

With all these standardized steps you can innovate, experiment, and focus on proposing new ideas in the field of metaheuristics supported by the No Free Lunch Theorem [17,18,19]. By implementing this framework, we envision a significant enhancement in the comparability and reliability of findings in this field, thereby contributing to its methodological rigor and practical applicability.

## Figures and Tables

**Table 1:** Comparison with other systematic literature reviews.

Paper	Year	Objective Function	Evaluation Metrics	Optimization Techniques	Classifier	Benchmark Application	Real-Word Application
[4]	2023	✓		✓	✓		
[5]	2023			✓			
[6]	2023		✓	✓	✓		✓
[7]	2023	✓	✓	✓	✓	✓	
[8]	2022			✓			
[9]	2022	✓		✓	✓		✓
[10]	2022	✓	✓	✓	✓	✓	
[11]	2022	✓		✓			✓
[12]	2022			✓	✓		
[13]	2021		✓	✓		✓	
[3]	2021		✓	✓	✓	✓	
[14]	2020	✓		✓	✓	✓	
Our Work		✓	✓	✓	✓	✓	✓

**Table 2:** Summary of database searches for feature selection literature (2019–2023).

Database	Query	#Result
• IEEE Xplore	(“Document Title”:“Feature Selection”) and Filters Applied: 2019–2023	2204
• ScienceDirect by Elsevier	Title field: “Feature Selection” and Year field: “2019–2023”	1388
• Scopus	TITLE (“feature selection”) AND PUBYEAR > 2018 AND PUBYEAR < 2024 AND (LIMIT-TO (LANGUAGE, “English”)	8812
• SpringerLink	Title field: “Feature Selection”	3006
• Web of Sciences	(TI=(“feature selection”)) AND (DT==(“ARTICLE” OR “REVIEW”) AND LA==(“ENGLISH”) AND PY==(“2023” OR “2022” OR “2021” OR “2020” OR “2019”))	4713
• Wiley	[Publication Title: “feature selection”] AND [Earliest: (01/01/2019 TO 04/20/2023)]	220

**Table 3:** Confusion matrix.

	Predicted Negative	Predicted Positive
Actual negative	TN	FP
Actual positive	FN	TP

**Table 4:** Classifier descriptions.

Classifier	Description
Adaptive Boosting (ADABOOST)	Ensemble technique adjusting weights on misclassified instances for improved accuracy.
Artificial Neural Network (ANN)	Model inspired by the brain, with interconnected neurons for data processing.
Decision Tree (DT)	Divides data into branches by evaluating feature values, arriving at decisions at each internal node, and assigning class labels to leaf nodes.
Decision Tree C4.5 (DT C4.5)	Refined algorithm dividing data based on features, selecting attributes via info gain, handling varied types, missing values, and pruning.
Decision Tree J48 (DT J48)	Improved C4.5 in Weka, selects attributes with info gain, handles varied attributes, missing data, and pruning.
Discriminant Analysis (DA)	Technique finding linear combinations of features for class separation and dimensionality reduction.
Extreme Gradient Boosting (XGBOOST)	Boosting algorithm that builds strong learners by focusing on instances with poor previous learner performance.
Extreme Learning Machine (ELM)	Single-hidden-layer neural network that randomly assigns weights and determines output weights analytically.
Fuzzy Classifier (FC)	Classifier using fuzzy logic to handle uncertainty in data.
Fuzzy Min–Max Neuronal Network (FMM)	Fuzzy system for classification, handling uncertainty using membership functions.
Gaussian Naive Bayes (GNB)	Naive Bayes variation assuming Gaussian distribution of feature values.
Growing Hierarchical Self-Organizing Map (GHSOM)	Neural-network-based algorithm for clustering and visualization of high-dimensional data.
K-Nearest Neighbor (k-NN)	Assigns labels based on the majority class of k nearest neighbors.
Kernel Extreme Learning Machine (KELM)	ELM variant using kernel methods for nonlinear classification in high-dimensional space.
Kstar Classifier (KSTAR)	Lazy learning algorithm classifying new instances based on closest neighbors.
Latent Dirichlet Allocation (LDA)	Generative model used for topic modeling in text data, revealing hidden topic structures.
Light Gradient Boosting (LightGBM)	Gradient boosting with histogram-based training for efficiency and accuracy.
Logistic Model Tree (LMT)	Decision tree with leaf nodes containing logistic regression models.
Logistic Regression (LR)	Linear model estimating the probability of binary classification.
Multi-Label KNN (ML-KNN)	Extends k-NN for multi-label classification, allowing instances to have multiple labels.
Multi-Label Naive Bayes (MLNB)	Naive Bayes extension for multi-label classification problems.
Multilayer Perceptron (MLP)	Neural network with multiple layers for complex nonlinear mappings.
Naive Bayes (NB)	Probabilistic classifier based on Bayes’ theorem, assuming feature independence.
Oblique Random Forest Heterogeneous (OblRF(H))	Variant of random forest using oblique splits for decision trees.
Optimum-Path Forest (OPF)	Pattern recognition algorithm constructing decision boundaries through graph-based approach.
Random Forest (RF)	Ensemble classifier that combines multiple decision trees to improve accuracy.
Standard Voting Classifier (SVC)	Ensemble technique combining classifier predictions through majority voting.
Support Vector Machine (SVM)	Finds a hyperplane to separate classes, maximizing the margin between them.

**Table 5:** Overview of the multi-objective algorithms.

Year	Ref.	Algorithm	Focus	Innovation	Validation
2023	[70]	MOAEOSCA	Botnet detection in IoT opposition-based learning	Hybridization of AEO and SCA; bitwise operations	Achieved acceptable accuracy in Botnet detection in IoT
2022	[68]	CMODE	Multi-objective optimization and crowding distance	Rank based on non-dominated sorting optimization algorithms	Outperformed six state-of-the-art multi-objective algorithms
2022	[69]	PSOMMFS	High-dimensional feature selection adaptive local search	Information entropy-based initialization	Improved quality of Pareto front
2022	[120]	MOHHOAC	Feature selection using HHO chaotic local search	Associative learning; grey wolf optimization	Effective feature selection on sixteen UCI datasets
2022	[172]	BChOA	Biomedical data classification operator for enhanced exploration	Two binary variants of ChOA; crossover	Effective feature selection on biomedical datasets
2021	[63]	NSGA-III	Multi-label data feature selection maximizing feature-label correlation	Incorporation of additional objectives	Outperformed other algorithms on eight multi-label datasets
2021	[64]	DAEA	Bi-objective feature selection in classification diversity-based selection method	Duplication analysis method	Superior performance on 20 classification datasets
2021	[65]	MOPSO-ASFS	High-dimensional feature selection particle selection mechanism	Adaptive penalty mechanism; adaptive leading	Enhanced performance on high-dimensional datasets
2021	[66]	MOBIFS	Multi-objective feature selection roulette wheel mechanism	Bacterial foraging optimization algorithm	Effective removal of redundant features
2021	[73]	MOBGA-AOS	Feature selection as a pre-processing technique five crossover operators	Adaptive operator selection mechanism	Outperformed other evolutionary multi-objective algorithms
2021	[74]	MOIA/D-FSRank	Feature selection in L2R clonal selection and mutation operators	Tchebycheff decomposition; elite selection strategy	Significant improvements on public LETOR datasets
2021	[179]	OBCOOA	Wrapper-based feature selection; opposition-based learning mechanism	Time-varying V-shape transfer function	Applied to 27 benchmark datasets
2020	[23]	MOSCA_FS	Hyperspectral imagery feature selection Jeffries–Matusita distance and mutual information	Novel discrete SCA framework; ratio between	Tested on diverse datasets
2020	[71]	BMOGW	Feature selection	Multi-objective grey wolf optimizer	Effective feature selection with reduced classification error rates
2020	[72]	BCNSG3 & BCNSG2	Multi-objective feature selection	Cuckoo optimization algorithm	Achieved non-dominated solutions with reduced error rates
2020	[175]	EGA	Early time-series classification mathematical model targeting classification performance	Emphasis on the starting time of classification	Outperformed a general genetic algorithm
2019	[62]	TMABC-FS	Cost-sensitive feature selection diversity-guiding searches; dual-archive system	Introduction of convergence and	Demonstrated robustness on UCI datasets

**Table 6:** This is a wide table.

Year	Ref.	Description	Field	Instances	Features	Classes	Repository
2018	[199]	Colon	Medical	62	2000	2	ASU
2001	[200]	SPECT Heart	Medical	267	22	2	UCI
1998	[201]	Dermatology	Medical	366	34	6	UCI
1994	[202]	Musk (Version 1)	Chemistry	476	166	2	UCI
1994	[203]	Breast Cancer Wisconsin (Diagnostic)	Medical	569	30	2	UCI
1992	[204]	Breast Cancer Wisconsin (Original)	Medical	699	9	2	UCI
1992	[205]	Lung Cancer	Medical	32	56	3	UCI
1991	[206]	Wine	Biology/Chemistry	178	13	3	UCI
1991	[207]	Tic-Tac-Toe Endgame	Game	958	9	2	UCI
1990	[208]	Zoo	Biology	101	16	7	UCI
1989	[209]	Ionosphere	Physical Science	351	34	2	UCI
1989	[210]	Chess (King-Rook vs. King-Pawn)	Game	3196	36	2	UCI
1988	[211]	Waveform Database Generator (Version 2)	Synthetic	5000	40	3	UCI
1988	[212]	Lymphography	Medical	148	18	4	UCI
1987	[213]	Congressional Voting Records	Politics	435	16	2	UCI
-	[214]	Sonar	Physical Science	208	60	2	UCI
-	[215]	Statlog heart	Medical	270	13	2	UCI
-	n.a.	Exactly	n.d.	1000	13	2	UCI
-	n.a.	Exactly2	n.d.	1000	13	2	UCI
-	n.a.	m-of-n	Biological	1000	13	2	UCI

**Table 7:** Curated repositories of datasets utilized in feature selection and optimization research.

Repository	Source	Use
AI Studio	https://aistudio.baidu.com/	Real-world application
ASU Repository (Scikit-feature Repository)	http://featureselection.asu.edu/datasets.php	Benchmark
AWID dataset	https://icsdweb.aegean.gr/awid/	Benchmark
BCI Competitions	https://www.bbci.de/competition/	Benchmark
Biopatrec Repository	https://github.com/biopatrec/biopatrec	Real-world application
Causality workbench	https://www.causality.inf.ethz.ch/	Benchmark
Dr. Wang’s Repository dataset	http://infosec.bjtu.edu.cn/wangwei/?page_id=85	Benchmark
Drug bank database	https://go.drugbank.com/	Real-world application
Github	https://github.com/	Benchmark
Kaggle	https://www.kaggle.com/	Benchmark
Keel	https://sci2s.ugr.es/keel/datasets.php	Benchmark
Ke Chen - Ph.D Candidate Datasets Repository	https://ckzixf.github.io/dataset.html	Benchmark
Mulan Library	https://mulan.sourceforge.net/	Benchmark
NCBI National Centre for Biotechnology Information	https://www.ncbi.nlm.nih.gov/	Real-world application
Near East Hospital	https://neareasthospital.com/	Real-world application
Ninapro	https://www.idiap.ch/project/ninapro/	Real-world application
Letor, By Microsoft	https://www.microsoft.com/en-us/research/	Benchmark
Papers with code	https://paperswithcode.com/	Benchmark
Physionet	https://physionet.org/	Real-world application
Quare.ai HeadCT Study	http://headctstudy.qure.ai/	Real-world application
RDRR	https://rdrr.io/r/utils/data.html	Benchmark
RSNA	https://www.rsna.org/	Real-world application
Time Series Machine Learning Website	https://www.timeseriesclassification.com/	Benchmark
UCI Repository	https://archive.ics.uci.edu/	Benchmark
UNB CIC Repository	https://www.unb.ca/cic/datasets/index.html	Benchmark
UNSW Repository	https://unsworks.unsw.edu.au/home	Benchmark
Yahoo	https://webscope.sandbox.yahoo.com/	Benchmark
Zexuan ZHU Professor Datasets Repository	https://csse.szu.edu.cn/staff/zhuzx/index.html	Benchmark

## Data Availability

Data are contained within the article.
